# A Predictive Toxicokinetic Model for Nickel Leaching from Vascular Stents

**DOI:** 10.1021/acsbiomaterials.3c01436

**Published:** 2024-03-25

**Authors:** Matheos Giakoumi, Pavlos S. Stephanou, Despoina Kokkinidou, Chara Papastefanou, Andreas Anayiotos, Konstantinos Kapnisis

**Affiliations:** † Department of Mechanical Engineering and Materials Science and Engineering, Cyprus University of Technology, Limassol 3036, Cyprus; ‡ Department of Chemical Engineering, Cyprus University of Technology, Limassol 3036, Cyprus; § Cp Foodlab Ltd, Nicosia 2326, Cyprus

**Keywords:** vascular stents, nickel leaching, mouse stent implantation model, physiologically based toxicokinetic (PBTK) models, multiobjective optimization, toxicological risk assessment

## Abstract

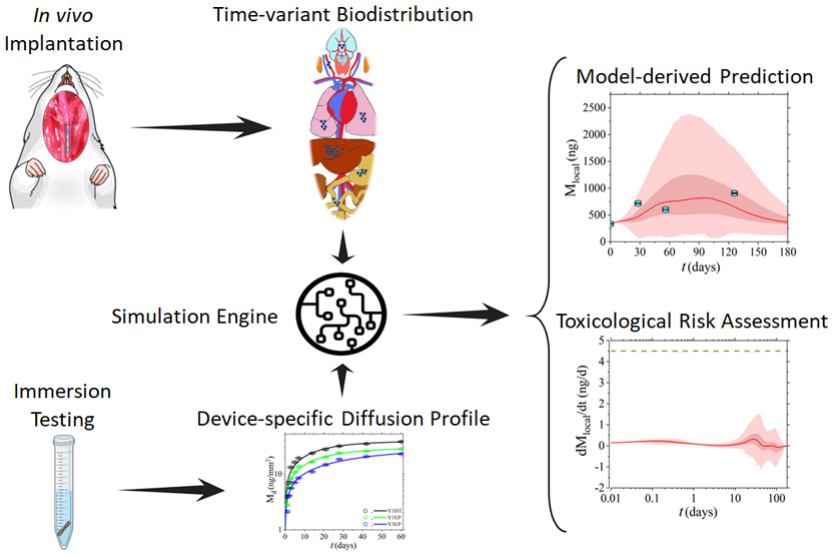

*In vitro* testing methods offer valuable insights into the corrosion vulnerability of metal implants and enable prompt comparison between devices. However, they fall short in predicting the extent of leaching and the biodistribution of implant byproducts under *in vivo* conditions. Physiologically based toxicokinetic (PBTK) models are capable of quantitatively establishing such correlations and therefore provide a powerful tool in advancing nonclinical methods to test medical implants and assess patient exposure to implant debris. In this study, we present a multicompartment PBTK model and a simulation engine for toxicological risk assessment of vascular stents. The mathematical model consists of a detailed set of constitutive equations that describe the transfer of nickel ions from the device to peri-implant tissue and circulation and the nickel mass exchange between blood and the various tissues/organs and excreta. Model parameterization was performed using (1) in-house-produced data from immersion testing to compute the device-specific diffusion parameters and (2) full-scale animal *in situ* implantation studies to extract the mammalian-specific biokinetic functions that characterize the time-dependent biodistribution of the released ions. The PBTK model was put to the test using a simulation engine to estimate the concentration–time profiles, along with confidence intervals through probabilistic Monte Carlo, of nickel ions leaching from the implanted devices and determine if permissible exposure limits are exceeded. The model-derived output demonstrated prognostic conformity with reported experimental data, indicating that it may provide the basis for the broader use of modeling and simulation tools to guide the optimal design of implantable devices in compliance with exposure limits and other regulatory requirements.

## 1 Introduction

In recent decades, there has been a significant surge in the use of implantable devices driven by not only a growing clinical demand but also rapidly evolving technologies in composite and synthetic materials science.^1,2^ The increasing incidence of chronic illnesses and the expanding elderly demographic are the primary drivers of the global medical implant market,^2^ and thus, continual research and development are required to improve their performance and extend their useful life span.^3^

Stents are cylindrically shaped, hollow mesh metal structures used to restore the flow in blood vessels and ducts.^4^ The corrosion resistance of implant materials results from optimal surface treatments during the manufacturing process, such as electropolishing, acid passivation, and protective coatings.^5−7^ Although this passivating film insulates the bulk material from the corrosive physiologic fluids, any mechanically induced break or defect in it increases the risk of corrosion.^5^ While metal alloys might endure significant forward and reverse mechanical strain excursions, the presence of a nonsuperelastic oxide layer could lead to cracking. This creates a pathway for the exposure of metal ion-rich phases to the *in vivo* environment, ultimately causing elevated ion release and reduced resistance to pitting.^5,8^ Experimental and clinical data, collectively reported by the U.S. Food and Drug Administration (FDA), suggest that metal implants experience different degrees of wear and corrosion due to the mechanical and biochemical environment at the specific site of implantation.^9^ For vascular (coronary and peripheral) stents, the *in vivo* setting, comprising complex vascular geometries and dynamic loading profiles, can affect corrosion susceptibility and result in surface damage or a change in ion diffusion kinetics.^10−16^ Allergenic, toxic/cytotoxic, or carcinogenic substances may be released into the body during the degradation processes, which contaminate the surrounding tissue and/or the bloodstream and may cause several adverse local as well as systemic effects.^17−20^ The majority of metal alloys used for stent manufacturing contain high levels of nickel, which is metallurgically necessary to impart enhanced mechanical properties but may lead to several adverse health effects if leached in high concentrations.^9^

To assess general corrosion susceptibility and metal ion release, the FDA recommendations include testing per the American Standard of Testing and Materials (ASTM) F2129-08 (Standard Test Method for Conducting Cyclic Potentiodynamic Polarization Measurements to Determine the Corrosion Susceptibility of Small Implant Devices),^21^ the ASTM F3306-19 (Standard Test Method for Ion Release Evaluation of Medical Implants),^22^ and the ASTM G31-72(2004) (Standard Practice for Laboratory Immersion Corrosion Testing of Metals).^23^ Various studies have investigated the corrosion resistance and metal ion release from nitinol stent-based devices.^24−26^ Specifically, Sullivan et al.^24^ studied the effect of oxide layer composition in nitinol stents manufactured by various processing methods and suggested strong correlations between the layer thickness and cumulative Ni release. Nagaraja et al.^26^ tested generic heart valve frames manufactured with different vacuum arc remelting methods and surface finishes and showed that the localized and uniform corrosion performance is maintained in higher microstructural nitinol purity.

In addition to providing a framework for comparing different alloys, designs, or manufacturing processes, this type of testing and data are used to estimate exposure as part of toxicological risk assessment per ISO 10993 (Biological Evaluation of Medical Devices).^27^ Despite the valuable insights that *in vitro* test methods can offer regarding the corrosion susceptibility of a particular device, these tests are usually carried out under idealized or hyperphysiological conditions. Consequently, even though these tests facilitate easy comparisons between devices, the degree to which *in vitro* performance aligns with *in vivo* corrosion behavior remains uncertain. Interestingly, Nagaraja et al.^28^ highlighted the impact of fatigue loading on uniform corrosion for different nitinol stent surfaces, while Sussman et al.^29^ demonstrated increased Ni release from nitinol devices exposed to different physiological environments characterized by the level of pH and reactive oxygen species (ROS). These findings emphasize the significance of taking the implantation site into account when designing studies to predict nickel release from medical implants and underscore the importance of the biomechanochemical environment at the device–tissue interface.

The FDA guidance document (Technical Considerations for Non-Clinical Assessment of Medical Devices Containing Nitinol Guidance for Industry and Food and Drug Administration Staff, issued on July 9, 2021)^30^ recommends that testing should be performed on as-manufactured devices and in simulated-use conditions. This type of testing should closely mimic physiologic conditions and capture both the initial bolus release of the substance and the longer-term release profile *in vitro*. Animal studies are often used to evaluate biocorrosion in a relevant anatomical site under simulated clinical conditions.^31^ However, biocompatibility assessments are not typically designed to evaluate the biological response to mechanical failure, and in some cases, anatomical differences between humans and animal models limit quantitative correlation. Additional studies are required to evaluate the *in vivo* response to failure modes such as coating defects and the release of wear particles and ions. Although linking the results of *in vitro* testing to *in vivo* outcomes is a formidable challenge, available data do suggest that there are at least qualitative consistencies. Data following *in situ* implantation of stents are scarce in the literature, yet Nagaraja et al.^32^ presented one of the very few studies that investigated the relationship between nitinol surface processing, *in vivo* nickel release, and biocompatibility. Nitinol stents manufactured using different surface treatments, and previously characterized for their *in vitro* release profile,^24^ were evaluated in a porcine stent implantation model. The investigation focused on the concentration of ions in local vascular tissue and in serum and urine samples and highlighted that those stents with nonoptimized surface finishing had significantly greater nickel leaching and gave rise to adverse inflammatory reactions and restenosis compared to polished stents.

While there is qualitative consistency between engineering testing and the behavior inside the body among devices, the quantification of these relationships remains a challenge for corrosion prediction within individual patients. The FDA suggests that a relatively easy and promising approach to obtain such quantitative relationships is the use of modeling and simulation tools.^9,30,33^ Physiologically based toxicokinetic (PBTK) models bear the capability to connect *in vivo* ion release with clinically measurable parameters such as ion concentrations in blood or urine. This allows for a direct comparison between the inferred *in vivo* exposure from these measurements and the outcomes of *in vitro* testing.^34^ Establishing such correlations necessitates a combination of computational modeling and *in vitro*, *ex vivo*, and *in vivo* testing and eventually clinical studies to parameterize and validate the model predictions. Although a number of studies have reported data regarding the concentration of different metal ions in serum and urine for healthy adults,^35−38^ there is still limited information about the levels of released ions, especially their accumulation in tissues/organs following the *in situ* implantation of medical devices.^39,40^ Biodistribution studies in animals are mostly after the injection^41,42^ or oral administration^43,44^ of metal ions, while the collection of toxicological data from implanted patients has many challenges associated with data heterogenicity and nonroutine follow-up examinations.

Saylor et al.^45^ proposed a biokinetic model that estimates nickel release from an implanted device. However, due to the lack of coherent data, the model was parameterized using immersion corrosion testing of nitinol stents^24^ and values following implantation of atrium occluders in humans.^46^ A recent work by some of us^47^ presented an expanded and enriched version of that model by adding a separate organ (kidney) compartment to better resemble normal physiology and by introducing time-dependent functions to describe some of the biokinetic parameters. The upgraded model was exercised in conjunction with probabilistic Monte Carlo simulations and exhibited quantitative consistency with nickel levels in serum and urine following the implantation of atrium occluders in humans^46^ and data reported by Nagaraja et al.^32^ on stent implantation in minipigs.

**Figure 1:**
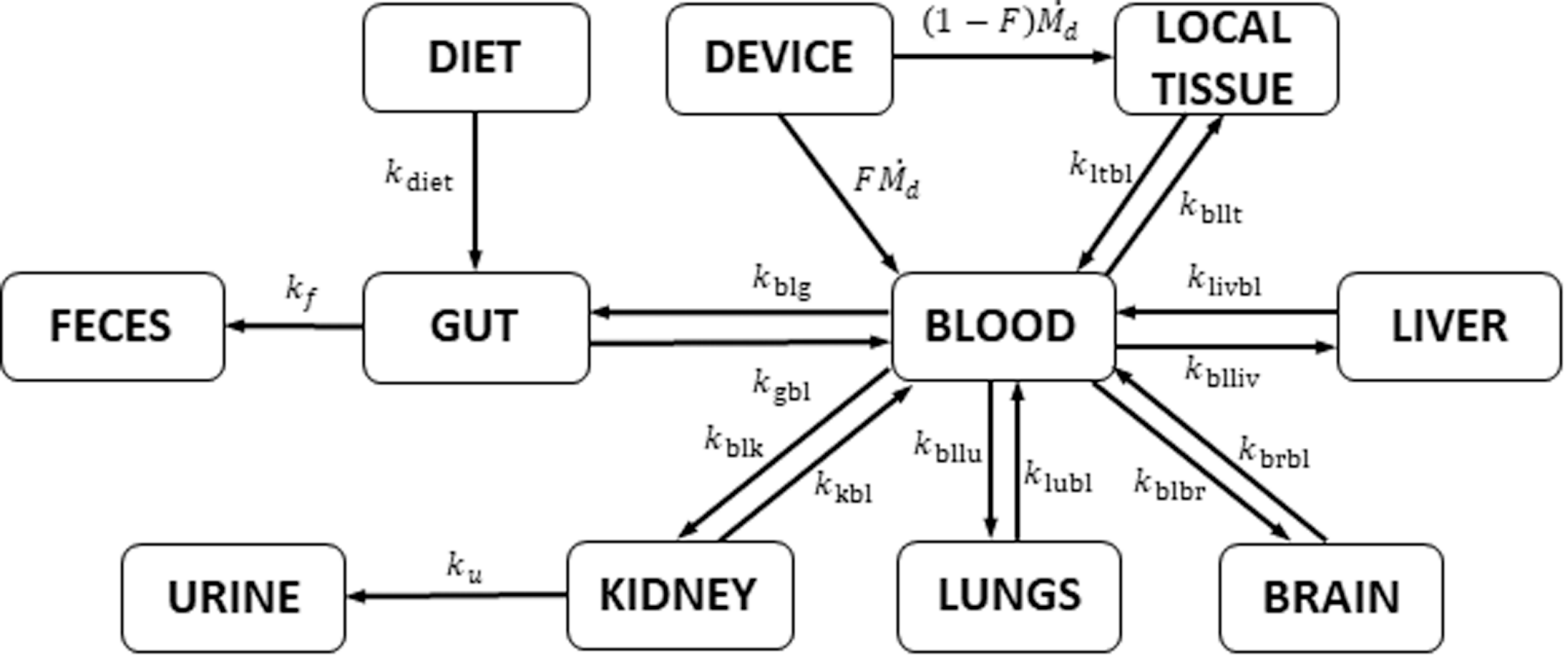
Diagram depicting the absorption, diffusion, distribution, and excretion principles of the proposed PBTK model.

The present work is, to the best of our knowledge, the first fully comprehensive study, which comprises device *in vitro* and *in vivo* testing, along with the formulation, parameterization, and testing of a multicompartment PBTK model and a simulation engine for toxicological risk assessment of vascular stents. The mathematical model describes the transfer of nickel ions from the device to adjacent tissue and circulation and the exchange between blood and the various tissues/organs (see Figure 1 and refer to Section 2.5.2). It is based on a detailed set of transport equations with the diffusion, absorption, distribution, and excretion variables as well as the appropriate initial and boundary conditions. Model parameterization was performed using (1) in-house-produced data from immersion testing to compute the device-specific diffusion parameters and (2) full-scale animal *in situ* implantation studies to extract the mammalian-specific biokinetic functions that characterize the time-dependent biodistribution of the released ions in tissues/organs, body fluids, and excreta. The developed *in silico* tool was then put to the test using a probabilistic Monte Carlo methodology to estimate the concentration–time profiles, along with confidence intervals, of nickel ions leaching from the implanted devices and determine if permissible exposure limits are exceeded.

## 2 Materials and Methods

### 2.1 Stent Manufacturing and Processing

**Figure 2:**
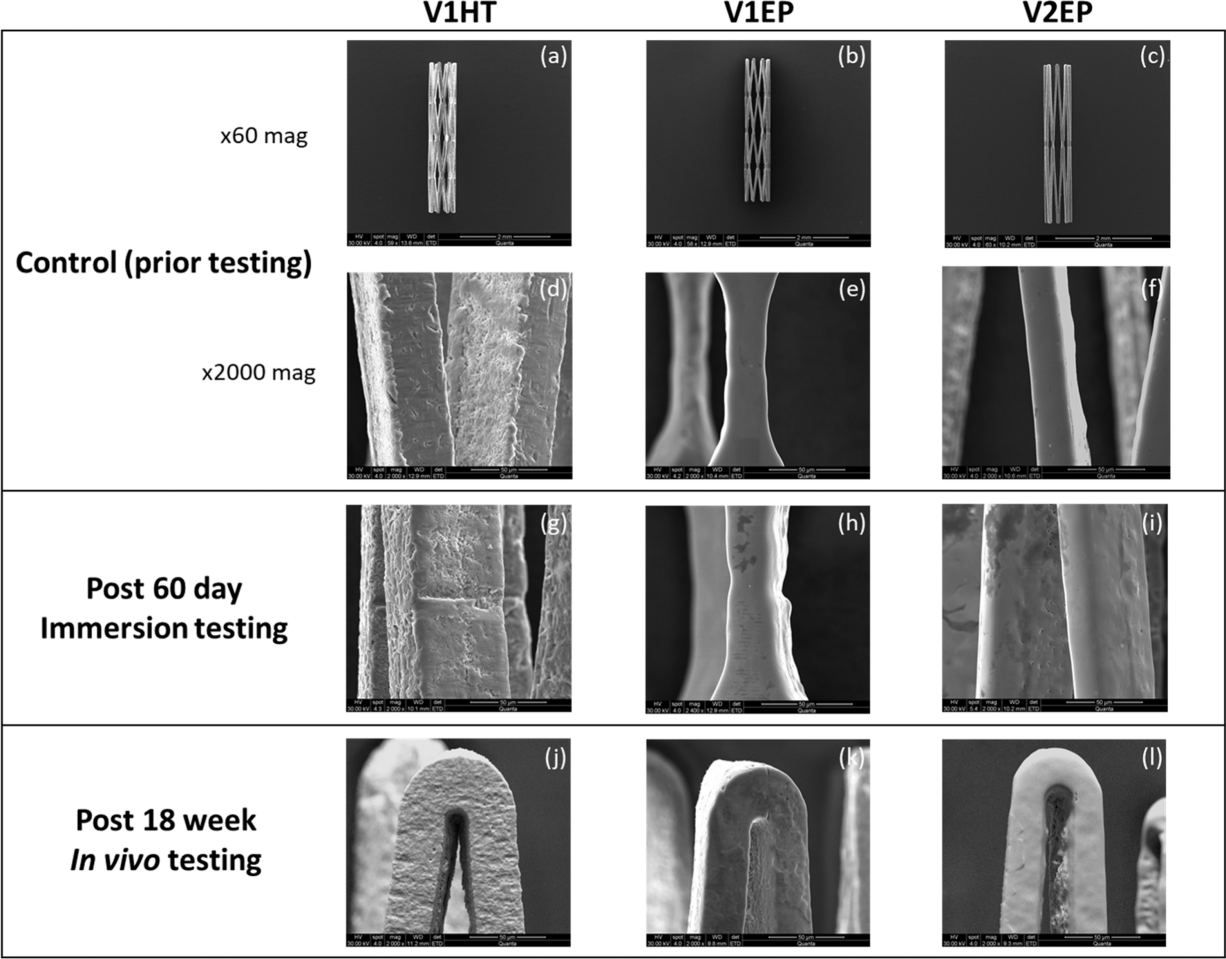
Representative scanning electron microscopy (SEM) images of control (prior testing) vs tested (*in vitro* and *in vivo*) stents. (a–c) The two stent designs, V1 and V2, after HT and EP surface processing (at ×60 mag). (d–f) Higher magnification (×2000) images showing inherent manufacturing features and imperfections in (d) HT stents. (g–i) Stent surface appearance and morphology post the 60 day immersion testing (at ×2000 mag). (j–l) Examples of different corrosion morphologies observed in explanted stents (at ×2000 mag).

Custom-made self-expanding nitinol stents (Admedes GmbH, Pforzheim, Germany), 0.7 mm × 3.3 mm in dimension, were used throughout the study. Two closed-cell designs with diamond-shaped patterns V1 (version 1—double cell) and V2 (version 2—single cell) (shown in Figure 2a–c) were fabricated to evaluate the effect of stress raisers (connective links/struts) on Ni ion release. After laser cutting, mechanical polishing was applied to eliminate the heat-affected zone from all stent samples. Subsequently, the stents were categorized into two groups based on material surface condition and processing steps—heat treatment (HT) and electropolishing (EP)—with a resulting strut thickness of approximately 40 and 20 μm, respectively. Heat treatment was employed to modify the metal surface chemistry and topography by creating a thicker titanium oxide layer and affecting the nickel-rich zone, thus acting as a source of high nickel release to mimic active *in vivo* corrosion. Electropolishing is the standard surface finishing method and was used to simulate a reduced level of ion leaching. These two groups cover a broad spectrum of potential manufacturing processes and help define a range of feasible release model parameters. Overall, we have studied three different devices: (1) the double-cell design, heat-treated stent (V1HT), (2) the double-cell design, electropolished stent (V1EP), and (3) the single-cell design, electropolished stent (V2EP).

### 2.2 Immersion Testing

All stent samples were weighted (*n* = 3 per group; 5-fold), and the exact values of their resulting volume and active surface area *A*_surf_ (per stent design/surface treatment method) were calculated based on the nitinol alloy density value (6.5 g/cm^3^) and three-dimensional (3D) model drawings (provided by the manufacturer).

Immersion testing was conducted on stent specimens (*n* = 3 per group; V1HT, V1EP, and V2EP), as per ASTM G31:72(2004)^23^ and ASTM F3306-19,^22^ to study the device-specific cumulative release profile data. All containers and instruments used for the testing, handling, and storage of specimens were acid-washed using a 10% HNO_3_ solution (100441 Supelco; Merck KGaA, Darmstadt, Germany) before use. Due to the small surface area of each device (∼11–12 mm^2^) and the low nickel concentrations in typical test solutions, sample pooling was performed for more accurate estimates. In each case, all 3 stents per group were placed in 15 mL polypropylene tubes filled with 10 mL of phosphate-buffered saline (PBS) (pH 7.4 ± 0.1), resulting in a PBS-volume-to-stent-surface-area ratio of 0.27–0.35 mL/mm^2^, depending on *A*_surf_ value. Although individual sample variability could not be analyzed with this method, a sufficient metal ion mass release (above the nickel detection limit of the analysis system) was required.^25^ During a 60-day incubation period at 37 °C, sampling was conducted at 10 time points (days 1, 2, 3, 5, 7, 14, 21, 28, 42, and 60) for nickel-ion measurements. At each time point, the total PBS volume was withdrawn from the test container, which was first flushed and then refilled with fresh solution.

The concentration of nickel ions was measured using high-resolution inductively coupled plasma mass spectrometry (ICP-MS) (XSERIES2, Elemental Scientific Inc., Omaha, NE). To monitor metallic contamination throughout all steps, nickel levels were also measured in *n* = 3 control blanks (PBS in tubes with no stent specimen). Samples were acidified with 2% HNO_3_ before analysis to ensure stability and comparability with calibration standards. The ICP-MS was tuned to the manufacturer’s recommendations and calibrated using blanks. Known standards were analyzed to create a calibration curve with a linearity greater than 0.999. Raw measurements were obtained in units of ppb (ng/mL).

### 2.3 *In Vivo* Testing

#### 2.3.1 Animal Implantation

Approval for all procedures involving animals was granted by the Cyprus Veterinary Services (project license no. CY/EXP/PR.L7/2018). The experiments were conducted at a fully licensed animal research laboratory (license no. CY.EXP.108) and in agreement with European and International guidelines (Directive 2010/63/EU of the European Parliament, National Institutes of Health (NIH) Guide for the Care and Use of Laboratory Animals). Male CD1 mice, weighing 45 ± 5 g (10–12 weeks old), chosen for their relatively large body size, were used throughout the study.

The three stent types (V1HT, V1EP, and V2EP) were tested *in vivo* to establish the time-dependent concentration of the released Ni ions in tissues, organs, and body fluids. Drug treatment and surgical procedures were carried out as per the reported experimental protocol by Kapnisis et al.,^48^ which describes in detail a method for stent implantation in mouse carotid artery. In brief, following general anesthesia, a small median incision is performed at the ventral neck area, and the left common carotid artery (LCCA) is exposed. The internal carotid artery (ICA) and LCCA are controlled, distally and proximally, respectively, by slings, and the external carotid artery (ECA) is ligated distally. Following blood flow interruption, a small incision is performed on the ECA and a polymeric catheter containing the stent is guided into the desired position in the LCCA. After stent deployment, with a stent-to-artery-size ratio range of 1.1–1.2, the wound is closed and the animals are allowed to recover.

#### 2.3.2 Nickel Biodistribution Analysis

Stented mice (*n* = 3 per stent type and time point) were euthanized under anesthesia at 4, 8, and 18 weeks (±1 day at each time point) after surgery. Harvested tissue/organs (stented arteries, kidneys, liver, lungs, brain, and small intestine) and whole blood, urine, and fecal samples were collected for nickel-ion quantification through ICP-MS. ICP-MS is the most commonly employed technique for detecting metal ions in biological samples and is the preferred analytical method due to its high sensitivity.^49^

Vascular tissue surrounding explanted stents was removed through a digestion process in a 1 M solution of NaOH, according to the protocol described in Kapnisis et al.^48^ Whole blood was collected via retro-orbital bleeding, and untainted urine specimens were collected via terminal direct bladder puncture using a glass Pasteur pipette. To avoid issues and variations arising from handling errors, the tissue-digested solution, the harvested organs, and the body fluid samples were collected and pooled (*n* = 3 per stent type and time point) for more accurate estimates. Tissue/organs and body fluid samples were also collected from healthy animals (*n* = 5 pooled) to establish baseline concentrations of nickel. All tools and containers utilized in both the tissue removal process and the handling of the explants were nonmetallic and acid-washed using a 10% HNO_3_ solution prior to use. Nickel levels were also estimated in mouse food and water to calculate the average daily dietary uptake. Biological specimens were digested using a closed-vessel microwave digestion procedure before analysis via ICP-MS as described in Section 2.2.

### 2.4 Stent Surface Evaluation

Following immersion testing and peri-implant tissue dissolution (for explanted devices), each stent sample was transferred into 1.5 mL Eppendorf tubes filled with ultrapure deionized water for 20 min of ultrasonication (twice) and allowed to air-dry overnight before surface evaluation. Scanning electron microscopy (SEM) analysis was performed prior to and post testing (*in vitro* and *in vivo*) using a Quanta 200 scanning electron microscope (FEI, Hillsboro, OR) to characterize and compare the surface topography and features of immersed and explanted to as-received nontested (control) stents. The abluminal (outer), laser-cut side wall, and luminal (inner) surfaces along the entire length of each sample were inspected for any signs of wear and/or corrosion. Elemental analysis, with energy-dispersive X-ray spectroscopy (EDS), was also performed (prior to and after testing) on the outer stent surfaces to test for altered elemental composition and quantify nickel-ion release upon active corrosion. The weight % ratio of nickel to titanium (Ni/Ti) was derived from the EDS spectra from tested and control stents of the same surface treatment group.

### 2.5 Model Formulation

#### 2.5.1 Device Release

By considering the physics-based diffusion model described by Giakoumi et al.,^47^ the device’s cumulative amount of nickel release (*M*_d_) per surface area (*A*_surf_) can be mathematically described as
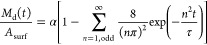
1with a rate of
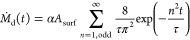
2where α represents the quantity of nickel connected to the surface per unit area, and τ denotes the characteristic release time.

#### 2.5.2 Biokinetics

Giakoumi et al.^47^ proposed a compartmental time-variant PBTK model for Ni leaching from cardiovascular stents by extending one proposed by Saylor et al.^45^ along with a method for determining the confidence intervals associated with the model predictions. The model demonstrated prognostic conformity with reported data following the implantation of nickel-containing cardiovascular devices in humans^46^ and minipigs^32^ in the compartments of serum, urine, and local tissue while providing nonvalidated predictions for the compartments of kidney and other tissues. However, the unavailability of measurements from other tissues and organs did not enable further validation and development of the model by separating additional compartments that may be required for the thorough toxicological evaluation of the device. In this work, we present a generalized version of the model proposed by Giakoumi et al.,^47^ wherein the other tissue compartment is separated into the liver, brain, lungs, gut, and fecal compartments (Figure 1). The model considers a zero-order rate of absorption of dietary nickel from the diet into the gut (*k*_diet_), an irreversible first-order elimination of Ni from the gut through feces (*k*_f_), an irreversible first-order elimination of Ni from the kidney to urine (*k*_u_), a reversible first-order exchange of Ni between blood and local tissues (*k*_ltbl_ and *k*_bllt_), blood and liver (*k*_livbl_ and *k*_blliv_), blood and brain (*k*_brbl_ and *k*_blbr_), blood and lungs (*k*_lubl_ and *k*_bllu_), blood and kidney (*k*_kbl_ and *k*_blk_), blood and gut *(k*_gbl_ and *k*_blg_), and the rate at which nickel is released from the medical device, (*Ṁ*_d_) (eq 2). A fraction of the nickel (0 ≤ *F* (*t*) ≤ 1) is directly released into the blood, whereas the residual nickel is released into the local tissue encircling the device. The evolution equations of the PBTK model are as follows
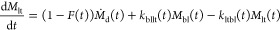
3a
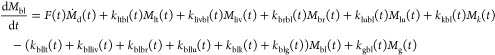
3b
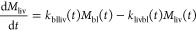
3c
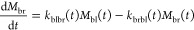
3d
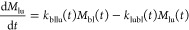
3e
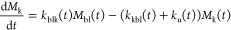
3f
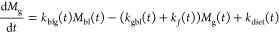
3g
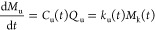
3h
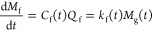
3iwhere *M*_lt_, *M*_bl_, *M*_liv_, *M*_br_, *M*_lu_*, M*_k_, *M*_g_, *M*_u_, and *M*_f_ are the nickel mass in the local tissue, whole blood, liver, brain, lungs, kidney, gut, urine, and fecal compartments, respectively. *C*_u_ and *C*_f_ correspond to the concentration of nickel in urine and feces, respectively, whereas *Q*_u_ and *Q*_f_ correspond to the volumetric urine and fecal output rates, respectively. Note that most of the kinetic rates are considered as time-dependent. The initial conditions for all of these quantities at *t* = 0 are set equal to their control/baseline values
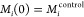
4This enables the calculation of the respective kinetic rates at *t* = 0
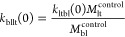
5a
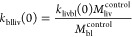
5b
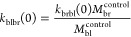
5c
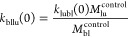
5d
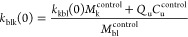
5e
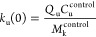
5f
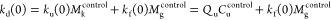
5g
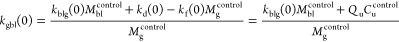
5hBy implementing non-negative, time-dependent kinetic rates, *k*_*i*_(*t*), along with a fraction *F*(*t*), the model can be considered as a time-variant system of ordinary differential equations (ODEs), for which in general no analytical solution exists, thus providing further flexibility for simulating the dynamics of Ni biodistribution in all of the compartments. Giakoumi et al.^47^ introduced a symmetric time-dependent function characterized by a Gaussian “pulse” behavior, expressed in the form of
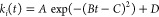
6awhere *A* (nickel mass/day) determines the pulse’s maximum height, *B* (1/day) is the pulse’s width, *C* is the abscissa of the saturation point, and *D* (nickel mass/day) is the kinetic rate’s steady-state value. Finally, fraction *F*(*t*) takes the form of
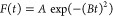
6bThe kinetic rate parameters *A*–*D* are treated as fitting parameters that can be optimized using the available *in vivo* experimental data, as discussed in Section 2.6. However, it should be noted that certain parameters can be directly calculated if the control values are experimentally available by utilizing eqs 5a–5h. It is important to highlight that in this particular case, when *t* = 0, only parameters *A, C*, and *D* can be calculated directly instead of being obtained through the fitting process. In this study, it was decided that it was more convenient to calculate the steady-state parameter (*D*) rather than the transient parameters *A* and *C* during the optimization procedure.

#### 2.5.3 Probabilistic Modeling

According to the methodology described by Giakoumi et al.,^47^ the implementation of probabilistic modeling techniques is considered more appropriate for evaluating the PBTK model prediction uncertainties associated with such a complicated system as the human body. This can be achieved by considering the fundamental physiological hypothesis of any PBTK model, which dictates that each kinetic rate must comprise the same values regardless of the device used but depending only on the mammalian-specific (device host) absorption, distribution, and excretion properties. Consequently, with the parameterization of the kinetic rates’ parameters for each device (see Section 2.6), it is possible to construct the probability density function (PDF) for each parameter.

The construction of the PDFs of the kinetic rates’ parameters, e.g., *f*_*Ak*_*i*__ for parameter *A* of the kinetic rate *k*_*i*_, can be achieved with the implementation of point estimation techniques, such as the maximum likelihood estimator (MLE),^50^ by considering each kinetic rate’s parameters as a random variable, e.g., ***A***, with observed values, e.g., *A*_1_, *A*_2_, ... , *A*_*n*_, in a random sample of size *n* (the number of tested devices). With the assumption that the kinetic rates’ parameters follow a log–normal or normal distribution based on the domain of the particular kinetic rates’ parameter, two unknown PDF’s parameters remain to be determined: the mean μ and the variance σ^2^ for a normal distribution (or the mean θ or variance ω^2^ for a log–normal distribution, the two types of distributions used in this paper), which are considered as optimization parameters that should maximize a function. This function, called MLE, takes the form of the likelihood function of the sample. For example, assuming that the parameter *A* of the kinetic rate *k*_*i*_ follows a normal distribution, the likelihood function is defined as *L*_*Ak*_*i*__(μ_*Ak*_*i*__, σ_*Ak*_*i*__^2^) = ∏_*n*_*f*_*Ak*_*i*__(*A*_*n*_, μ_*Ak*_*i*__, σ_*Ak*_*i*__^2^). The likelihood functions are maximized at the maximum likelihood estimators μ̂_*Ak*_*i*__ and σ̂_*Ak*_*i*__^2^.

With the determination of the estimators for all of the kinetic rates’ parameters and the construction of their PDF, the PBTK model can be solved using probabilistic methods, such as the Monte Carlo (MC) probabilistic method,^51^ where each kinetic rate parameter’s value is randomly drawn from its PDF through a series of random virtual experiments (virtual experiments that result in a negative concentration in any compartment at any time are interrupted and discarded). This will produce the biodistribution profile in various tissues/organs and body fluid compartments, which can be depicted in a Whisker plot featuring the median value, the interquartile range (IQR), and the upper–lower Whisker range (maximum = *Q*3 + 1.5 × IQR and minimum = *Q*1–1.5 × IQR) of the data.^50^

### 2.6 Model Parameterization: Optimization

For the determination of the kinetic rates’ parameters for each tested device, an optimization problem must be constructed. In this work, we choose to use as the objective function the root-mean-square error (RMSE) between the prediction of the PBTK model (*M*_*i*_*(t))* and the experimental data *j* (*j* ∈ [1, *N*_*i*_], where *N*_*i*_ is the total number of experimental data in compartment *i*). Since the solution of the PBTK model provides the prediction for each compartment *i*, with a total of *P* compartments (*P* ≥ 2), and assuming that there are experimental data available for each compartment, then the objective function can be considered as a multiobjective function, defined as , with , where **k** is the vector that includes the kinetic rates’ parameters (*A*, *B*, *C*, *D*) and the *F*(*t*) parameters (*A*, *B*) to be optimized (as defined by Giakoumi et al.^47^), and **K** is the feasible set of **k**. Note that the optimal value of **k** is the one that aligns with the solution’s Pareto front, of the multiobjective optimization problem, as determined by a preference function.

One way is to define the preference function as implemented by Giakoumi et al.^47^ Briefly, by encoding the preferences as a vector of weights^52^ and by using the weighted sum method,^53^ the multiobjective function is converted to a single objective function. Since currently there is no indication in the literature regarding the importance of any specific compartment relative to the rest of the compartments, the same trade-off weight factors^52,53^ can be used, redefining the optimization problem as
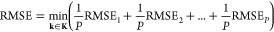
8As such, we may now use the single objective optimization algorithm patternsearch from the Global Optimization Toolbox available in MATLAB.^54^ Note that eq 8 can be interpreted as the average error of the model output and the experimental data in units of mass and can thus be used to assess the “goodness” of the fit or to compare the various predictions along different devices.

The feasible set **K**(47) is determined by the domain and linear inequalities related to the kinetic rates’ parameters as
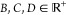
9a
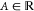
9b
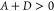
9cwhereas for the fraction *F*(*t*), the domain and linear inequalities are given as
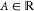
9d
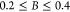
9eFor the establishment of a well-defined objective function, it is important to acknowledge the possibility of having experimental data in the acquired sample that, due to the complexity of their acquisition, are sources of experimental errors. To account for such errors, we assign a weight factor to every experimental data point *j* in compartment *i* (*w*_*ij*_), indicating the level of confidence in the data. A numerical value for these weight factors can be assigned by considering limiting conditions, such as

(1) The Ni mass in any compartment cannot be less than its control value (*M*_*i*1_), described as *M*_*ij*_ ≥ *M*_*i*1_,*j* > 1.
(2) The Ni mass in any compartment cannot exceed the total cumulated release of the device *M*_d_ (eq 1) plus the control value, described as *M*_*ij*_ ≤ *M*_*d*_(*t*_*j*_) + *M*_*i*1_, *j* > 1.

If these conditions are met, then a 100% confidence is assigned (*w*_*ij*_ = 1); otherwise, a confidence less than 100% must be assigned, which in this study is quantified by using a Gaussian radial basis function (RBF).^55^ The RBF is a kernel-type function that quantifies the similarities between data. In this work, we choose
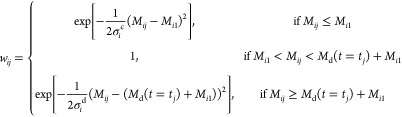
10where
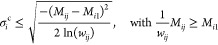
11a
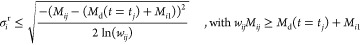
11bThe parameter σ_*i*_^c^ refers to the violation of the control value (first limiting condition), and the parameter σ_*i*_^r^ refers to the violation of the total cumulated device release (second limiting condition). If these parameters are not preset, they can be estimated by considering the equalities in eqs 11a and 11b after determining *w*_*ij*_ (e.g., from eq 11a*w*_*ij*_ = *M*_*ij*_/*M*_*i1*_, which defines the maximum percentage reduction on the experimental data *j* in the compartment *i* to stop violating the limiting conditions). However, note that there is only one parameter σ_*i*_ for each compartment *i*, and therefore if more than one data violates the conditions, then the determined *w*_*ij*_ will not have the same σ_*i*_ parameter value despite referring to the same compartment. In this situation, the optimized σ_*i*_ is derived through an optimization procedure similar to the one applied for the *in vitro* parameters (with the starting point of the optimization procedure to be the mean of the different resulting σ_*i*_ parameters in compartment *i*) and then applied to recalculate the *w*_*ij*_ of these data via eq 10. Therefore, the level of confidence for each data defined by eq 10 can be included in the objective function defined in eq 8 through the redefinition of RMSE_*i*_
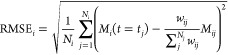
12where the term *∑*_*j*_^*N*_*j*_^*w*_*ij*_ normalizes the data among compartments.

Finally, according to Giakoumi et al.,^47^ the *in vitro* parameters of eq 2 can be obtained by a standard nonlinear optimization algorithm.

### 2.7 Tolerable Intake Analysis

We have recently^47^ proposed a method for performing a tolerable intake (TI) analysis based on FDA recommendations^27^ of a TI value for parenteral (nonoral) exposure to nickel of 0.5 μg/kg/day.^27,30^ As per the method, the device’s nickel release (eq 2) and release rate profile predicted in each compartment (d*M*_*i*_*(t)*/d*t*) are evaluated against the TI value for nickel to assess if the design and physicochemical characteristics of a device are toxicologically safe or unsafe. We hypothesized that the TI value per compartment can be evaluated via TI_*i*_ = ⟨*y*_*i*_⟩ × 0.50 μg/(kg.day), where ⟨*y*_*i*_⟩ = ⟨*M*_*i*_⟩/⟨*M*_total_⟩ represents the time-averaged mass fraction of nickel in the *i*^th^ compartment, which can be estimated stochastically using available experimental data and the mass balance equation. For the newly proposed model (Figure 1), the following mass balance equation can be defined by considering the control/baseline mass values, the Ni diet intake, and the total devise release
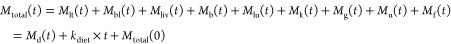
13

## 3 Results

### 3.1 Stent Surface Characterization and Ni Ion Quantification

Stent surface processing resulted in visually different colors for the two types (HT vs EP), indicating unique oxide layers. HT stents were dark in appearance, whereas EP stents had a bright, shiny metallic appearance. According to the manufacturer, the resulting oxide layer thickness could range between 5 and 100 nm for EP- and HT-treated surfaces, respectively. Yet, the samples’ surface topography did not allow us for any precise oxide layer thickness measurements, neither via X-ray reflectometry (XRR) nor ultraviolet–visible (UV–vis) spectroscopy (data not shown).

Nontested (control) HT stents had inherent manufacturing surface features and imperfections that pre-existed the *in vitro* and/or the *in vivo* testing (see Figure 2d), while EP stents had smooth consistent surfaces throughout. The evaluation also confirmed that the surface roughness decreases after the various processing steps, from HT to EP (Figure 2d–f). Next, the 60 day immersion testing revealed no evidence of significant uniform corrosion for any group when compared to preimmersion samples (see Figure 2g–i). On the contrary, explanted stents from mice, HT, in particular, displayed rougher surfaces predominately on the abluminal (outer) part compared to their nonimplanted controls (see Figure 2j–l). Some microcracks were observed with “bubbling” of the oxide indicating potential subsurface corrosion (see Figure 2j). In any case, the EDS analysis did not expose any differences in the Ni/Ti ratio between tested (immersed or explanted) and nontested (control) stents since the levels of Ni ion leach were typically below the 1–2% by weight EDS detection limit (data not shown).

**Figure 3:**
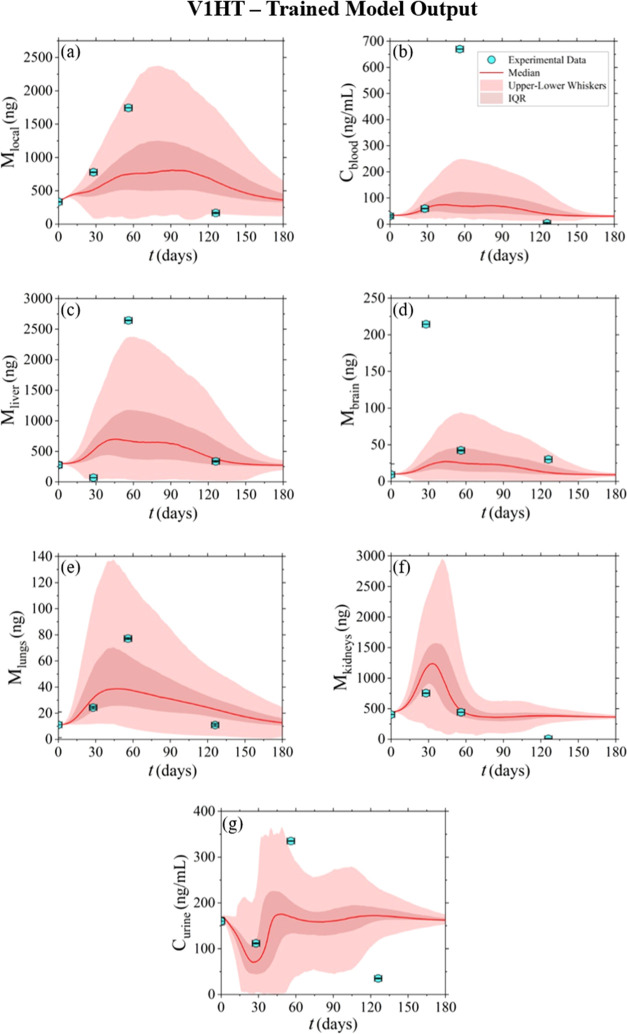
Model-derived output of the nickel concentration–time profiles, released from V1HT stents, in the various tissue/organ and body fluid compartments.

**Figure 4:**
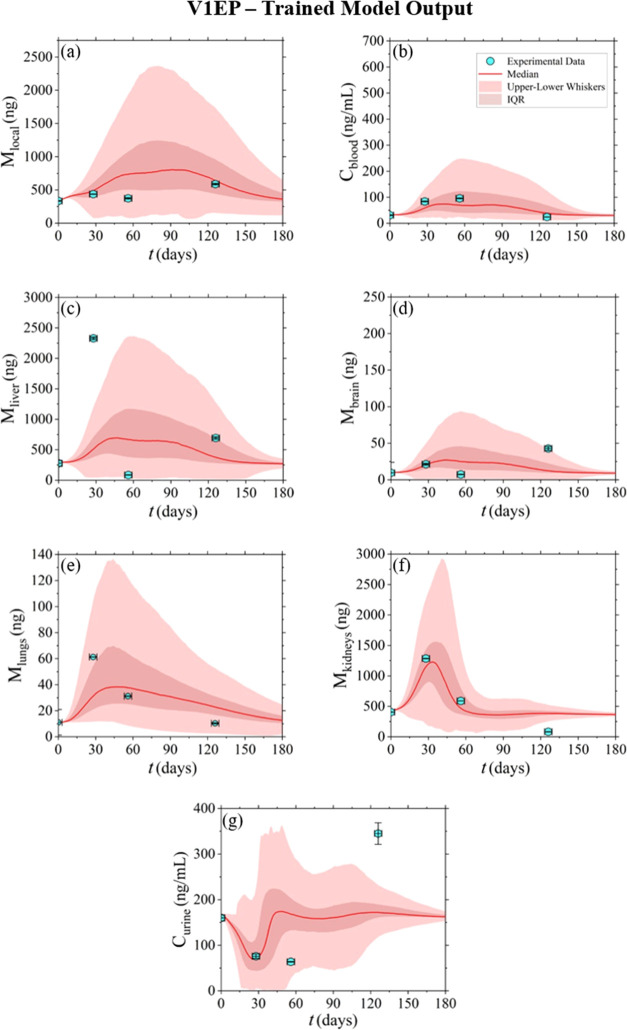
Model-derived output of the nickel concentration–time profiles, released from V1EP stents, in the various tissue/organ and body fluid compartments.

**Figure 5:**
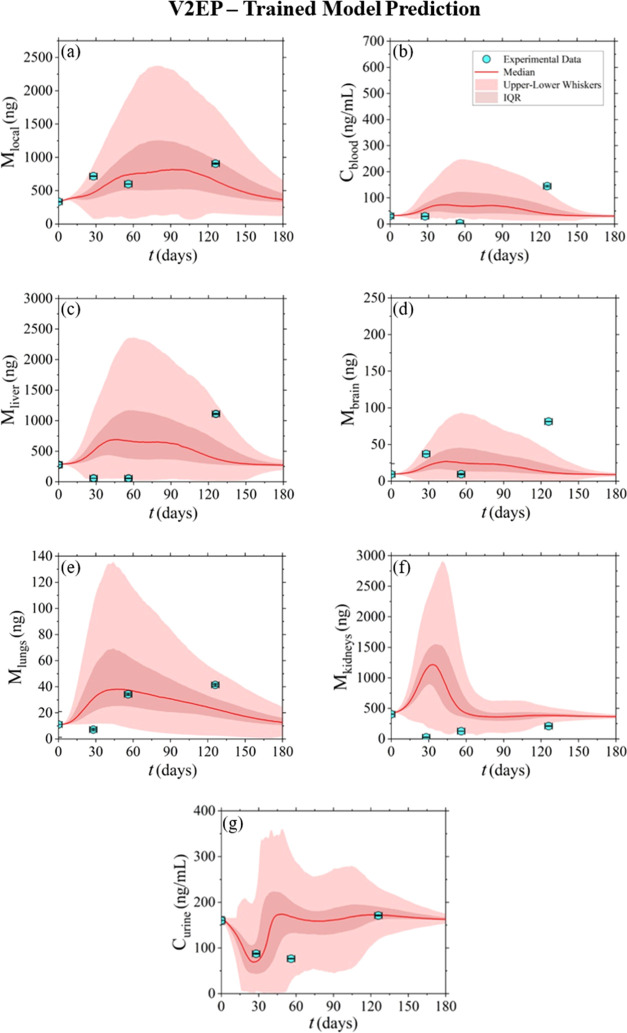
Model-derived prediction of the nickel concentration–time profiles, released from V2EP stents, in the various tissue/organ and body fluid compartments.

The concentration of nickel ions in all test solutions was quantified through ICP-MS. The resulting detection limit for Ni was below 1 ppb, and the reproducibility observed after triplicate measurement per sample resulted in low standard deviation values. Nickel levels in control blanks (PBS in tubes with no stent specimen) averaged 5.7 ppb and were deducted from all measurements from immersion testing. The cumulative Ni release profile for the V1HT, V1EP, and V2EP devices, following the 60-day immersion testing, is shown in Figure S1. The detected concentration in each step was multiplied by the solution volume per specimen (i.e., 3.33 mL) to calculate the total mass of nickel (in ng) leached from each device. Regardless of the geometric design (V1 or V2), HT stents exhibited the greatest amount of Ni release throughout 60 days of immersion (Figure S1), confirming that electropolishing is a much more effective surface processing method for controlling ion leaching from metallic biomaterials. Interestingly, the V1 design demonstrated higher cumulative release than the V2 design when comparing the EP-treated stents. This is most probably because the double-cell diamond-shaped pattern (V1) results in a larger number of stress raisers (connective links and struts) that promote stress-induced corrosion. Nickel levels were measured in harvested tissue/organs (stented arteries, kidneys, liver, lungs, brain, and small intestine), blood, urine, and fecal samples and were processed to determine the time-dependent concentration profile of implant-leached ions in the mammalian living system (see Figures 3–5). In general, HT stents demonstrated a significantly higher Ni ion release, most notably in the peri-implant tissue, compared to EP stents with varying biodistribution patterns over the 18-week implantation period. These data were subsequently used to extract the device-specific release parameters and the mammalian-specific biokinetic parameters during the model training phase and also to validate the model-derived predictions during the prognostic phase.

### 3.2 Application of the Model

The methodology described in Sections 2.5.3 and 2.6 was employed to parameterize the developed PBTK model, using the *in vitro* immersion data (indicated in Figure S1) and the *in vivo* biodistribution measurements (indicated in Figures S2 and S3), thus enabling the generation of probabilistic time-dependent profiles for nickel levels across the various model compartments. The predictive value of the PBTK model was tested and validated against experimental data (independent training and testing sets) in an attempt to provide a straightforward link between device release characteristics and ion concentration–time profiles in tissues/organs, body fluids, and excreta.

The fitting process and model parameterization using the resulting *in vitro* and *in vivo* data can be found in the Supporting Information. Measurements from the V1HT and V1EP devices were used for model training, while data from the evaluation of the V2EP stent type were used as the evaluation (testing) subset. The physiological parameters considered for a 45 g CD1 mouse (Section 2.3.1) were as follows: *V*_b_ = 3.6 mL,^56^*Q*_u_ = 1 mL/day,^57^*V*_u_ = 0.15 mL,^58^*Q*_f_ = 0.094 g/day (in-house estimation), and *k*_diet_ = 1250 ng/d (in-house estimation). The fitted results for devices V1HT and V1EP are depicted in Figures S2 and S3, respectively. We have omitted graphs for the gut and fecal compartments due to high dispersion in the experimental data but mainly because the detected mass did not differ significantly from the control values *(M*_g_^control^ = 2180 ng and *C*_f_^control^ = 11 593 ng/mL) and was attributed merely to dietary intake and not the device release.

Figures 3 and 4 present the model-derived estimates after conducting 5000 Monte Carlo experiments for devices V1HT and V1EP, respectively. Each parameter was derived from its PDF, as described in Section 2.5.3. It is important to note that these outcomes are not predictive in the dimension of α and τ, as the sampling was performed using parameters trained from the same data set. Nonetheless, the results demonstrate a highly satisfactory description provided by the probabilistic model, as the majority of the data points fall within the uncertainty range, most notably for the V1EP device. This can be observed by the RMSE of each device and for each compartment, as presented in Table 1. The median is represented by red continuous lines, the interquartile range (IQR) is depicted with a dark red shading, and the upper–lower whisker range is represented by a lighter red shading. Furthermore, it is worth mentioning that the data points falling within the numerical uncertainty range are consistent with the resulting weight factors calculated based on Section 2.6, as presented in Table S2 of the Supporting Information.

**Table 1:** Root-Mean-Square Error of Each Compartment (RMSE_*i*_) for the Median Output of the Probabilistic PBTK Model for the Devices V1HT, V1EP, and V2EP

	RMSE_*i*_ (eq 12)		
compartment	V1HT	V1EP	V2EP
local tissue (ng)	145.2	43.9	73.3
blood (ng)	179.7	32.7	44.1
liver (ng)	178.9	188.6	250.2
brain (ng)	32.5	5.9	12.1
lungs (ng)	7.0	5.5	5.1
kidneys (ng)	99.9	44.4	285.7
urine (ng)	5.5	5.1	2.3
(eq 8)	92.7	46.6	96.1

Figure 5 illustrates the predictions obtained by the model, along with a comparison against the *in vivo* data from V2EP, which were not included in the data set used for training the model. While the prediction does not exhibit the same level of satisfaction as for the previous devices, it is noteworthy that the majority of the data points that fall outside the uncertainty range were detected below the control/baseline levels. Consequently, these data points do not indicate a violation of toxicological safety, suggesting that deviations from the expected range are acceptable.

### 3.3 Toxicological Risk Assessment

The PBTK model-derived outputs were employed to assess whether nickel exposure levels exceed permissible limits and determine the toxicological safety of implants with different surface finishes. In Figure S4 (see the Supporting Information), the time-dependent device release rate (*Ṁ*_d_), was compared to the total TI value, revealing that all devices surpass the predefined exposure threshold for approximately the first 20–70 h after implantation.

**Figure 6:**
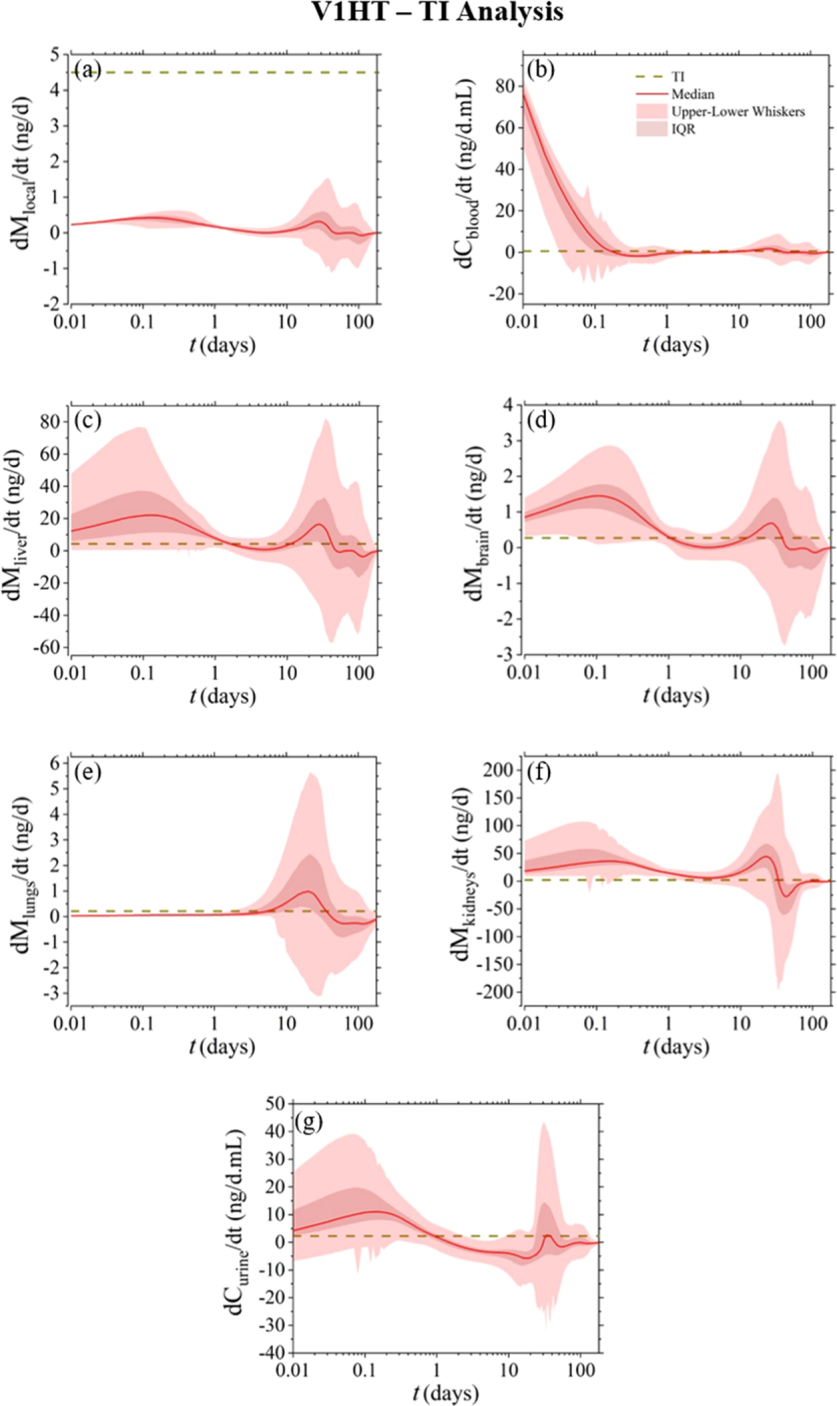
Model-derived compartmental release rates (d*M*_*i*_/d*t*) for V1HT. To enable toxicological risk assessment, the horizontal dashed line corresponds to the compartmental (depicted based only on the median *y*_*i*_ value obtained from 5000 Monte Carlo computational experiments) TI_*i*_ per body mass, as defined in Section 2.7.

**Figure 7:**
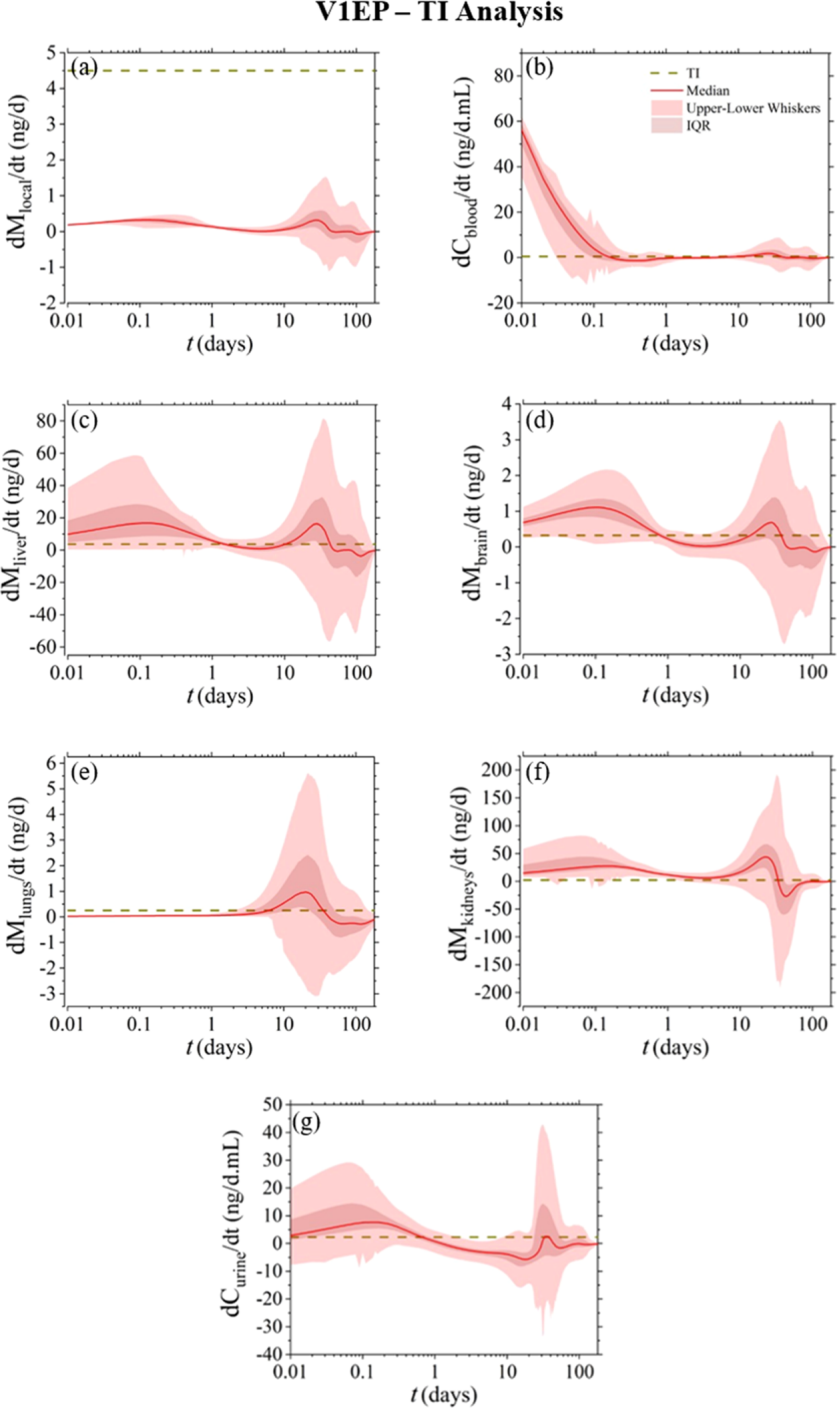
Model-derived compartmental release rates (d*M*_*i*_/d*t*) for V1EP. To enable toxicological risk assessment, the horizontal dashed line corresponds to the compartmental (depicted based only on the median *y*_*i*_ value obtained from 5000 Monte Carlo computational experiments) TI_*i*_ per body mass, as defined in Section 2.7.

**Figure 8:**
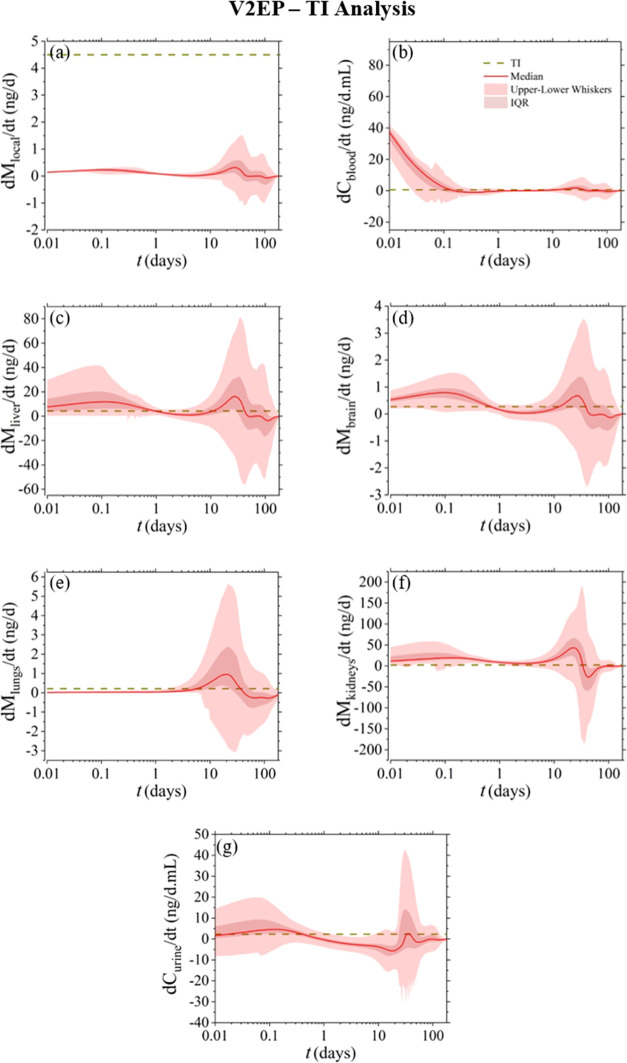
Model-derived compartmental release rates (d*M*_*i*_/d*t*) for V2EP. To enable toxicological risk assessment, the horizontal dashed line corresponds to the compartmental (depicted based only on the median *y*_*i*_ value obtained from 5000 Monte Carlo computational experiments) TI_*i*_ per body mass, as defined in Section 2.7.

For evaluating the toxicological exposure in individual tissue/organs and body fluid compartments, the release rate profiles *(dM*_*i*_/*dt)* for all compartments in the model were compared against the compartmental TI_*i*_ values, as defined in Section 2.7. Figures 6–8 present the results of this analysis for the V1HT, V1EP, and V2EP devices, respectively, which exhibit similar profiles, with the only discernible difference being an increased response and wider numerical uncertainty before day 1 for the V1HT and V1EP devices.

When focusing the evaluation on each separate compartment, local tissue is the only compartment that in all cases does not exceed the permissible limits. On the other hand, the nickel release rate in the blood exceeds the permissible limits during the first few hours post implantation and over again after day 10, with higher values denoted for the V1HT device. The liver, brain, and kidney compartments exhibit similar behavior as in blood but with the early-stage threshold violation expanding until day 1. Conversely, the lung compartment reveals a toxicological risk between days 5 and 30, whereas urine increases a concern only within the first 24 h, with the level and timespan of the predicted violations decreasing between the V1HT, V1EP, and V2EP devices.

## 4 Discussion

The degradative behavior of implant materials has been extensively investigated in the framework of quality assurance, failure analysis, and implant retrieval analysis and is considered a mandatory step in the regulatory approval process.^59−63^ The use of computational modeling and simulation (CM&S) in regulatory submissions is rapidly increasing and together with bench, nonclinical *in vivo*, and clinical studies can also be used to evaluate the toxicological safety and effectiveness of medical devices.^64,65^ The FDA^66^ and the EU Reference Laboratory for Alternatives to Animal Testing (EURL ECVAM)^67^ promote and facilitate the use of nonanimal methods in testing and research that have the potential to provide timelier and more predictive information to assess certain aspects of regulated products.

For many years, CM&S studies have been employed to support engineering design and structural analysis^68−70^ as they have the potential to streamline the development process and alleviate challenges related to premarket device evaluation. However, CM&S can also enhance the information content from traditional *in vitro* or *in vivo* assessments, such as unexpected biomechanical adverse effects that go undetectable within a study sample but occur frequently enough within a target population.^71−73^ To ensure the accuracy and precision of results for supporting regulatory submissions, it is essential to develop processes and approaches that foster consistency in the execution and review of CM&S.^33,74^ While prior art provides the means of modeling the diffusion of substances in mammalian compartments and comprehensively considers the release from implantable devices,^45,75−80^ it does not provide a practical and cost-effective way of guiding the optimal design of implantable devices to ensure compliance with a permissible exposure limit of different mammalian compartments to concentrations of released substances including intervals of confidence. There is therefore the need for a robust and validated modeling technology that overcomes the above-mentioned limitations so that device manufacturers can optimize device design and ensure that the release of substances will remain below acceptable thresholds.

Herein, a multicompartment PBTK model is presented along with a probabilistic Monte Carlo methodology for toxicological risk assessment of vascular stents. In this study, we have modified the PBTK model proposed by Giakoumi et al.,^47^ considering the major organs (brain, lungs, and liver) associated with a toxicological concern as well as blood and excreta (urine and feces) as separate compartments. To the best of our knowledge, this is the very first study that reports on the *in vivo* concentration of nickel ions in all of these compartments. The PBTK model was trained (parameterized) by using only a subset of the available *in vivo* data (those obtained using the devices V1HT and V1EP; Figures 3 and 4) and inputs using *in vitro* nickel release measurements; the trained model was then employed to make a prediction for a third device (V2EP, Figure 5) following the leave-one-out cross-validation (LOOCV) approach.^81^

Although the latter prediction (V2EP) did not fully meet the specified performance requirements after the training model, it was found to be in good agreement with the majority of the data points that lie beyond the uncertainty range. These are the ones below the control level, which are expected to be erroneous and can also explain why the RMSE of V2EP is very similar to that of V1HT. The physical limitations of the data below the control level play a detrimental role here, resulting in smaller possible disparities between the prediction profile and the outlier data. In contrast, the difference between the prediction profile and the outlier data, which exceeds the total cumulative release, appears to have no limitations and is therefore expected to have significantly higher disparities. Consequently, employing RMSE as a quantitative measure for the model’s predictive performance may not be precise in this scenario, underscoring the necessity for future studies to investigate more representative methods to evaluate the prediction performance numerically.

The optimization procedure presented in this work represents a significant improvement over the previous methodology,^47^ primarily due to the availability of experimental data in all compartments, allowing for a more comprehensive development of the methodology. However, this advancement has also increased the complexity of the optimization procedure, especially by incorporating a larger sample of experimental data from different compartments that are also subjected to experimental errors and uncertainties. In such cases, having a robust and well-defined objective function becomes crucial for the effective guidance of the implemented algorithm toward the desired basin of attraction points while avoiding stagnation at irrelevant points that do not represent the physiology of the organism under consideration. To simplify the optimization process and address the impact of experimental uncertainties, we take two key steps. First, we transform the model from a multiobjective function to a single objective function^52^ by considering an equalization of the importance of each compartment with weight factors (eq 8)^53^ and therefore providing a much more simplifying objective function for the algorithm to handle. Second, the methodology accounts for experimental errors in the sample data by integrating them as an intrinsic part of the objective function using kernel-type function^55^ (eq 10), which imparts smoothness to the objective function. The fact that the kinetic rates are considered time-dependent also provides enhanced flexibility to handle *in vivo* data from various mammals even in cases of contradictory behavior between the experimental measurements.

In Section 3.2, Figures 3 and 4 demonstrate that the integration of experiment errors as an intrinsic part of the objective function, based on eq 10 and Table S2 in the Supporting Information, was a successful implementation, with the majority of data points falling within the numerical uncertainty range consistently aligned with the resulting weight factors. However, in certain compartments, like the brain, significant dispersion in data among the devices was observed, not entirely consistent with the high-value weight factors. This discrepancy arises from high dissimilarities between data from devices V1HT and V1EP in the brain compartment, affecting the fitted parameters *k*_brbl_ and *k*_blbr_. Nonetheless, the model’s accurate fit to the brain compartment, as shown in Figures S2d and S3d of the Supporting Information, supports the overall success of the implementation of the weight factors in the optimization procedure.

The methodology presented here can be further applied to increase the level of confidence in experimental data from *in vitro* testing setups using a probabilistic stochastic (Monte Carlo) methodology. Nagaraja and Pelton^25^ studied the nickel elution resistance of electropolished nitinol ocular microstents via immersion testing and showed that the cumulative nickel release profiles of different sets of the same tested samples varied over a 63-day immersion duration. Predictive stochastic simulations could be used as an additional tool in regulatory testing to estimate the median values and the dispersion from multiple experiments and therefore upgrade the credibility of standard guides for the corrosion and elution resistance of medical implants. It is important to emphasize that the proposed methodology is generic and applicable to any simulation engine that requires training with hard-to-obtain data characterized by high uncertainty and experimental errors, providing a valuable approach for modeling complex systems under such conditions. We note that it should be possible to extend the framework presented in this paper to other device applications and metal ions. For instance, cobalt–chromium alloys, widely utilized in both vascular and orthopedic device applications, could be considered, provided there is adequate data for parameterization and, if needed, adjustment of the suggested multiscale model.

As made evident from the literature survey, this is the first fully comprehensive study that reports on a multicompartment PBTK model that includes the most important tissues/organs and the necessary *in vivo* data to parameterize it. Other studies, for simplicity, have employed PBTK models that include only very few of the organs in separate compartments, and all of the rest are included in an “other tissues” compartment. This then poses the question of whether our multicompartment PBTK model can be “simplified” by lumping together some tissue compartments. It can be easily shown mathematically that this can indeed be done (see the Supporting Information) since the time-averaged mass fractions of the tissue compartments that are lumped are known. However, the reverse (splitting) cannot be done even if the time-averaged mass fractions are assumed. This strongly exemplifies the necessity to acquire measurements from as many organs and tissues as possible.

Herein, the model outputs aligned with existing *in vivo* data, indicating the potential use of *in vitro* nickel release testing for estimating both local and systemic exposure. Yet, a constraint of the current model lies in assuming a static local implant environment. The local biomechanical environment plays a decisive role in the degradative performance of an implant.^28^ The complex and varying *in vivo* settings comprising dynamic geometries, cyclic and dynamic loading profiles, and harsh chemical conditions (chloride, dissolved oxygen, and pH levels) can affect the corrosion susceptibility that results from surface damage or a change in ion diffusion kinetics. Hence, it is crucial to comprehend and assess the diverse interactions that an implantable device is anticipated to undergo *in vivo* in order to identify the requisite testing for establishing a reasonable assurance of safety.

In addition, vascular devices undergo dynamic physiological changes, owing to inflammation and gradual tissue coverage. This leads to alterations not only in local biochemistry but also in the evolving geometry at the implant site over time. As the device becomes enveloped by the endothelium, the initially assumed model geometry, which considered nitinol wires to be only partially embedded in peri-implant tissue, is no longer a valid approximation. However, incorporating the rate of coverage, as now captured by *F*(*t*), facilitates a more accurate consideration of the impact of this process on local nickel accumulation within the model. Moreover, in certain cases, peri-implant tissue nickel concentrations are more sensitive to release and may surpass local thresholds for toxic effects, even at relatively low release rates compared to the chronic threshold for typical device sizes. In other words, the variability in blood nickel levels within the patient population may obscure the detection of nickel release, which could cause adverse effects locally. This also implies that local effects due to nickel release are more likely than systemic effects in most vascular device applications. Nonetheless, it should be emphasized that the results presented in this study were based on a specific device geometry assumption.

Future work will emphasize advancing the technology using inputs from a larger animal model, like swine, which is a well-established model, that will allow us to resolve size-related issues that previously hindered sample collection and detection limits. Data from a wide range of potential manufacturing processes, in terms of bulk material, geometric design, total active surface area, and surface processing methods, are required for extensive model parameterization. A wide data set will also facilitate the implementation of perturbation theory and machine learning (PTML) methods early in the device design cycle to optimize device-specific characteristics to minimize ion leaching. Large animal models offer important translational features and the ability to apply human-like settings, which increases the chances of bench findings being translated into effective clinical tools. Species-specific physiology between the different laboratory animals and further on to human trials is, of course, challenging because of the uncertain comparability of physiological processes. Different knowledge-driven approaches for considering physiological and biochemical differences between them should be evaluated by systematically incorporating specific model parameter domains of a target species into the PBTK model of a reference species. Cross-species extrapolation and ultimately full model validation with coherent clinical data will enable such novel technology to exploit the increased market acceptance and benefit fully from the entry into this rapidly expanding sector.

## 5 Conclusions

A novel multicompartment and time-variant physiologically based toxicokinetic (PBTK) model was developed and parameterized with *in vitro* immersion tests and *in vivo* implantation studies. The model predictions were complemented with a probabilistic Monte Carlo approach and a simulation engine to estimate the concentration–time profiles, along with confidence intervals, of implant-leached ions in all of the major organs associated with a toxicological concern as well as in peri-implant tissue, body fluids, and excreta. However, more robust *in vivo* implantation studies from large animals are required to further improve the feasibility and reliability of the model. Despite its limitations, the proposed model may provide the basis for the broader use of prognostic tools to guide the optimal design of implantable devices in compliance with exposure limits and other regulatory requirements.

## Data Availability

The underlying codes for this study (and training/validation data sets) are not publicly available for proprietary reasons (International Application No. PCT/EP2022/064468).

## Supporting Information Available

Optimization scheme employed in obtaining the cumulative nickel release parameters of devices V1HT, V1EP, and V2EP, as designated in eq 1; the results of parameterizing those devices on the PBTK model outlined in eqs 3a–3i; the toxicological risk assessment based on the device release rate; and the lumping and splitting discussion of the PBTK model (PDF)

## Notes

The authors confirm that they have given due consideration to the protection of intellectual property associated with this work (Patent Cooperation Treaty (PCT) WO/2022/248703) and that there are no impediments to publication, including the timing of publication, with respect to intellectual property. In so doing, the authors confirm that they have followed the regulations of their institution concerning intellectual property. The authors also declare that they have no known competing financial interests or personal relationships that could have appeared to influence the work reported in this paper.

## References

[ref1] Agrawal A. A.; Pawar K. A.; Ghegade V. N.; Kapse A. A.; Patravale V. B. Nanobiomaterials for Medical Devices and Implants. Nanotechnol. Med. Biol. 2022, 235–272. 10.1016/B978-0-12-819469-0.00008-3.

[ref2] Precedence Research. Medical Implants Market Size to Surpass USD 173.41 Bn by 2032. https://www.precedenceresearch.com/medical-implants-market.

[ref3] Harman M. K. Medical Device Failure—Implant Retrieval, Evaluation, and Failure Analysis. Biomater. Sci. 2020, 1485–1495. 10.1016/B978-0-12-816137-1.00096-9.

[ref4] Scafa Udrişte A.; Niculescu A.-G.; Mihai Grumezescu A.; Bădilă E.; Vitoria B.; Ciurana Gay J.; Eceiza Mendiguren A.; Jesús Quiles Carrillo L. Cardiovascular Stents: A Review of Past, Current, and Emerging Devices. Materials 2021, 14 (10), 2498 10.3390/MA14102498. 34065986 PMC8151529

[ref5] Eliaz N. Corrosion of Metallic Biomaterials: A Review. Materials 2019, 12 (3), 407 10.3390/ma12030407. 30696087 PMC6384782

[ref6] Asri R. I. M.; Harun W. S. W.; Samykano M.; Lah N. A. C.; Ghani S. A. C.; Tarlochan F.; Raza M. R. Corrosion and Surface Modification on Biocompatible Metals: A Review. Mater. Sci. Eng., C 2017, 77, 1261–1274. 10.1016/j.msec.2017.04.102. 28532004

[ref7] Kapnisis K.; Constantinou M.; Kyrkou M.; Nikolaou P.; Anayiotos A.; Constantinides G. Nanotribological Response of A-C:H Coated Metallic Biomaterials: The Cases of Stainless Steel, Titanium, and Niobium. J. Appl. Biomater. Funct. Mater. 2018, 16 (4), 230–240. 10.1177/2280800018782840. 29974806

[ref8] Chen Q.; Thouas G. A. Metallic Implant Biomaterials. Mater. Sci. Eng., R 2015, 87, 1–57. 10.1016/j.mser.2014.10.001.

[ref9] Food and Drug Administration. Biological Responses to Metal Implants; FDA, 2019. www.fda.gov.

[ref10] Kapnisis K. K.; Pitsillides C. M.; Prokopi M. S.; Lapathitis G.; Karaiskos C.; Eleftheriou P. C.; Brott B. C.; Anderson P. G.; Lemons J. E.; Anayiotos A. S. In Vivo Monitoring of the Inflammatory Response in a Stented Mouse Aorta Model. J. Biomed. Mater. Res., Part A 2016, 104 (1), 227–238. 10.1002/jbm.a.35560. 26362825

[ref11] Wang J.; Jin X.; Huang Y.; Ran X.; Luo D.; Yang D.; Jia D.; Zhang K.; Tong J.; Deng X.; Wang G. Endovascular Stent-Induced Alterations in Host Artery Mechanical Environments and Their Roles in Stent Restenosis and Late Thrombosis. Regen. Biomater. 2018, 5 (3), 177–187. 10.1093/rb/rby006. 29942650 PMC6007795

[ref12] Chaabane C.; Otsuka F.; Virmani R.; Bochaton-Piallat M. L. Biological Responses in Stented Arteries. Cardiovasc. Res. 2013, 99 (2), 353–363. 10.1093/cvr/cvt115. 23667187

[ref13] Halwani D. O.; Anderson P. G.; Brott B. C.; Anayiotos A. S.; Lemons J. E. Clinical Device-Related Article Surface Characterization of Explanted Endovascular Stents: Evidence of in Vivo Corrosion. J. Biomed. Mater. Res., Part B 2010, 95B (1), 225–238. 10.1002/jbm.b.31698. 20737558

[ref14] Halwani D.; Anderson P.; Lemons J.; Jordan W.; Anayiotos A.; Brott B. In-Vivo Corrosion and Local Release of Metallic Ions from Vascular Stents into Surrounding Tissue. J. Invasive Cardiol. 2010, 22, 528–535. 21041849

[ref15] Kapnisis K. K.; Halwani D. O.; Brott B. C.; Anderson P. G.; Lemons J. E.; Anayiotos A. S. Stent Overlapping and Geometric Curvature Influence the Structural Integrity and Surface Characteristics of Coronary Nitinol Stents. J. Mech. Behav. Biomed. Mater. 2013, 20, 227–236. 10.1016/j.jmbbm.2012.11.006. 23313643

[ref16] Kapnisis K.; Constantinides G.; Georgiou H.; Cristea D.; Gabor C.; Munteanu D.; Brott B.; Anderson P.; Lemons J.; Anayiotos A. Multi-Scale Mechanical Investigation of Stainless Steel and Cobalt–Chromium Stents. J. Mech. Behav. Biomed. Mater. 2014, 40, 240–251. 10.1016/j.jmbbm.2014.09.010. 25255419

[ref17] Barchowsky A. Systemic and Immune Toxicity of Implanted Materials. In Biomaterials Science: An Introduction to Materials in Medicine; Academic Press, 2020; pp 791–799 10.1016/B978-0-12-816137-1.00051-9.

[ref18] Gori T. Vascular Wall Reactions to Coronary Stents—Clinical Implications for Stent Failure. Life 2021, 11 (1), 63 10.3390/LIFE11010063. 33477361 PMC7829777

[ref19] Nordberg G. F.; Costa M. Handbook on the Toxicology of Metals: Vol. I: General Considerations; Elsevier, 2021. 10.1016/B978-0-12-823292-7.01001-9.

[ref20] Matusiewicz H.; Richter M. Local Release of Metal Ions from Endovascular Metallic Implants in the Human Biological Specimens: An Overview of in Vivo Clinical Implications. World J. Adv. Res. Rev. 2021, 11 (1), 091–102. 10.30574/WJARR.2021.11.1.0326.

[ref21] American Society of Testing and Materials. ASTM F2129, Standard Test Method for Conducting Cyclic Potentiodynamic Polarization Measurements to Determine the Corrosion Susceptibility of Small Implant Devices. https://www.astm.org/f2129-08.html.

[ref22] American Society of Testing and Materials. ASTM F3306–19. Standard Test Method for Ion Release Evaluation of Medical Implants. https://www.astm.org/f3306-19.html.

[ref23] American Society of Testing and Materials. ASTM G31–72(2004). Standard Practice for Laboratory Immersion Corrosion Testing of Metals. https://www.astm.org/g0031-72r04.html.

[ref24] Sullivan S. J. L.; Dreher M. L.; Zheng J.; Chen L.; Madamba D.; Miyashiro K.; Trépanier C.; Nagaraja S. Effects of Oxide Layer Composition and Radial Compression on Nickel Release in Nitinol Stents. Shape Memory Superelasticity 2015, 1 (3), 319–327. 10.1007/s40830-015-0028-x.

[ref25] Nagaraja S.; Pelton A. R. Corrosion Resistance of a Nitinol Ocular Microstent: Implications on Biocompatibility. J. Biomed. Mater. Res., Part B 2020, 108 (6), 2681–2690. 10.1002/jbm.b.34599. 32159908

[ref26] Nagaraja S.; Sena G.; Stafford P.; Braeuner C.; Hempel P.; Pelton A. R.; Ravi V. Effects of Nitinol Microstructural Purity on Localized and Uniform Corrosion Susceptibility. Shape Memory Superelasticity 2022, 8 (2), 118–128. 10.1007/s40830-022-00366-1.

[ref27] Food and Drug Administration. *Use of International Standard ISO 10993–1*, “Biological Evaluation of Medical Devices - Part 1: Evaluation and Testing within a Risk Management Process”; FDA, 2020.

[ref28] Nagaraja S.; Chandrasekar V.; Ormonde D.; Hickey H.; Lipschultz K.; Chao C.; Vilendrer K.; Pelton A. R. The Impact of Fatigue Testing and Surface Processing on Nickel Release in Nitinol Stents. Shape Memory Superelasticity 2018, 4 (4), 462–471. 10.1007/s40830-018-00196-0.

[ref29] Sussman E. M.; Shi H.; Turner P. A.; Saylor D. M.; Weaver J. D.; Simon D. D.; Takmakov P.; Sivan S.; Shin H. Y.; Di Prima M. A.; Godar D. E. Nitinol Release of Nickel under Physiological Conditions: Effects of Surface Oxide, PH, Hydrogen Peroxide, and Sodium Hypochlorite. Shape Memory Superelasticity 2022, 8 (2), 98–106. 10.1007/s40830-022-00364-3. 37720627 PMC10502700

[ref30] Food and Drug Administration. Technical Considerations for Non-Clinical Assessment of Medical Devices Containing Nitinol; FDA; 2021.

[ref31] Sullivan S. J. L.; Madamba D.; Sivan S.; Miyashiro K.; Dreher M. L.; Trépanier C.; Nagaraja S. The Effects of Surface Processing on In-Vivo Corrosion of Nitinol Stents in a Porcine Model. Acta Biomater. 2017, 62, 385–396. 10.1016/j.actbio.2017.08.029. 28842334

[ref32] Nagaraja S.; Sullivan S. J. L.; Stafford P. R.; Lucas A. D.; Malkin E. Impact of Nitinol Stent Surface Processing on In-Vivo Nickel Release and Biological Response. Acta Biomater. 2018, 72, 424–433. 10.1016/j.actbio.2018.03.036. 29597023

[ref33] Food and Drug Administration. Assessing the Credibility of Computational Modeling and Simulation in Medical Device Submissions; FDA, 2021.

[ref34] Chen M.; Du R.; Zhang T.; Li C.; Bao W.; Xin F.; Hou S.; Yang Q.; Chen L.; Wang Q.; Zhu A. The Application of a Physiologically Based Toxicokinetic Model in Health Risk Assessment. Toxics 2023, 11 (10), 874 10.3390/toxics11100874. 37888724 PMC10611306

[ref35] Saravanabhavan G.; Werry K.; Walker M.; Haines D.; Malowany M.; Khoury C. Human Biomonitoring Reference Values for Metals and Trace Elements in Blood and Urine Derived from the Canadian Health Measures Survey 2007–2013. Int. J. Hyg. Environ. Health 2017, 220 (2), 189–200. 10.1016/j.ijheh.2016.10.006. 27776932

[ref36] Templeton D. M.; Sunderman F. W.; Herber R. F. M. Tentative Reference Values for Nickel Concentrations in Human Serum, Plasma, Blood, and Urine: Evaluation According to the TRACY Protocol. Sci. Total Environ. 1994, 148 (2–3), 243–251. 10.1016/0048-9697(94)90400-6. 8029699

[ref37] Genchi G.; Carocci A.; Lauria G.; Sinicropi M. S.; Catalano A. Nickel: Human Health and Environmental Toxicology. Int. J. Environ. Res. Public Health 2020, 17 (3), 679 10.3390/ijerph17030679. 31973020 PMC7037090

[ref38] Sunderman F. W.; Hopfer S. M.; Sweeney K. R.; Marcus A. H.; Most B. M.; Creason J. Nickel Absorption and Kinetics in Human Volunteers. Exp. Biol. Med. 1989, 191 (1), 5–11. 10.3181/00379727-191-42881. 2717626

[ref39] Elkiran O.; Karakurt C.; Kocak G.; Taskapan C. Serum Nickel and Titanium Levels after Transcatheter Closure of Atrial Septal Defects with Amplatzer Septal Occluder. Cardiol Res. Pract 2019, 2019. 1 10.1155/2019/7891746. PMC6334312 30719342

[ref40] Høl P. J.; Bell K.; Mølster A.; Gjerdet N. R. Nickel Contamination from an Intravenous Catheter Used for Infusion. Scand. J. Clin. Lab. Invest. 2005, 65 (3), 221–225. 10.1080/00365510510013578. 16095051

[ref41] Gregus Z.; Klaassen C. D. Disposition of Metals in Rats: A Comparative Study of Fecal, Urinary, and Biliary Excretion and Tissue Distribution of Eighteen Metals. Toxicol. Appl. Pharmacol. 1986, 85 (1), 24–38. 10.1016/0041-008X(86)90384-4. 3726885

[ref42] Magaye R. R.; Yue X.; Zou B.; Shi H.; Yu H.; Liu K.; Lin X.; Xu J.; Yang C.; Wu A.; Zhao J. Acute Toxicity of Nickel Nanoparticles in Rats after Intravenous Injection. Int. J. Nanomed. 2014, 9 (1), 1393–1402. 10.2147/IJN.S56212. PMC3958504 24648736

[ref43] Spears J. W.; Jones E. E.; Samsell L. J.; Armstrong W. D. Effect of Dietary Nickel on Growth, Urease Activity, Blood Parameters and Tissue Mineral Concentrations in the Neonatal Pig. J. Nutr. 1984, 114 (5), 845–853. 10.1093/jn/114.5.845. 6726454

[ref44] Kohiyama M.; Chimi K.; Kanematsu H.; Miki K.; Takahashi Y.; Shimizu M.; Niiya I.; Sugano M.; ku K. Tissue Incorporation and Excretion of Dietary Nickel in Rats. J. Jpn. Oil Chem. Soc. 1994, 43 (4), 357–363. 10.5650/jos1956.43.357.

[ref45] Saylor D. M.; Adidharma L.; Fisher J. W.; Brown R. P. A Biokinetic Model for Nickel Released from Cardiovascular Devices. Regul. Toxicol. Pharmacol. 2016, 80, 1–8. 10.1016/j.yrtph.2016.05.019. 27208438

[ref46] Burian M.; Neumann T.; Weber M.; Brandt R.; Geisslinger G.; Mitrovic V.; Hamm C. Nickel Release, a Possible Indicator for the Duration of Antiplatelet Treatment, from a Nickel Cardiac Device in Vivo: A Study in Patients with Atrial Septal Defects Implanted with an Amplatzer Occluder. Int. J. Clin. Pharmacol. Ther. 2006, 44 (3), 107–112. 10.5414/CPP44107. 16550732

[ref47] Giakoumi M.; Stephanou P. S.; Kapnisis K.; Anayiotos A. On the Development of Physiologically Based Toxicokinetic (PBTK) Models for Cardiovascular Implants. Regul. Toxicol. Pharmacol. 2023, 144, 105489 10.1016/j.yrtph.2023.105489. 37659713

[ref48] Kapnisis K.; Stylianou A.; Kokkinidou D.; Martin A.; Wang D.; Anderson P. G.; Prokopi M.; Papastefanou C.; Brott B. C.; Lemons J. E.; Anayiotos A. Multilevel Assessment of Stent-Induced Inflammation in the Adjacent Vascular Tissue. ACS Biomater. Sci. Eng. 2023, 9, 4747 10.1021/acsbiomaterials.3c00540. 37480152 PMC10428095

[ref49] Jose Chirayil C.; Abraham J.; Mishra R. K.; George S. C.; Thomas S. Instrumental Techniques for the Characterization of Nanoparticles. Therm. Rheol. Meas. Techn. Nanomater. Charact. 2017, 3, 1–36. 10.1016/B978-0-323-46139-9.00001-3.

[ref50] Montgomery D. C.; Runger G. C. Applied Statistics and Probability for Engineers, seventhth ed.; John Wiley & Sons, Inc, 2011.

[ref51] de Rocquigny E.; Devictor N.; Tarantola S. Uncertainty in Industrial Practice: A Guide to Quantitative Uncertainty Management; John Wiley & Sons, Ltd, 2008 10.1002/9780470770733.

[ref52] Kochenderfer M. J.; Wheeler T. A. Algorithms for Optimization; The MIT Press, 2019; p 520.

[ref53] Zadeh L. A. Optimality and Non-Scalar-Valued Performance Criteria. IEEE Trans. Automat. Control 1963, 8 (1), 59–60. 10.1109/TAC.1963.1105511.

[ref54] The MathWorks Inc. MATLAB Version: 9.14.0.2239454 (R2023a). Natick, Massachusetss 2023. https://www.mathworks.com/.

[ref55] Schölkopf B.; Tsuda K.; Vert J.-P.. Kernel Methods in Computational Biology; The MIT Press, 2004 10.7551/MITPRESS/4057.001.0001.

[ref56] O’Connell K. E.; Mikkola A. M.; Stepanek A. M.; Vernet A.; Hall C. D.; Sun C. C.; Yildirim E.; Staropoli J. F.; Lee J. T.; Brown D. E. Practical Murine Hematopathology: A Comparative Review and Implications for Research. Comp Med. 2015, 65 (2), 96–113. 25926395 PMC4408895

[ref57] Coleman C.; Fuller J.; Green M.; Kaliss N.; Russel E.; Staats J. Biology of the Laboratory Mouse; Green E., Ed.; Dover Publications: New York, 1966.

[ref58] Reis L. O.; Sopena J. M. G.; Fávaro W. J.; Martin M. C.; Simão A. F. L.; dos Reis R. B.; de Andrade M. F.; Domenech J. D.; Cardo C. C. Anatomical Features of the Urethra and Urinary Bladder Catheterization in Female Mice and Rats. An Essential Translational Tool. Acta Cir. Bras. 2011, 26 (SUPPL. 2), 106–110. 10.1590/S0102-86502011000800019. 22030824

[ref59] Amerstorfer F.; Fischerauer S. F.; Fischer L.; Eichler J.; Draxler J.; Zitek A.; Meischel M.; Martinelli E.; Kraus T.; Hann S.; Stanzl-Tschegg S. E.; Uggowitzer P. J.; Löffler J. F.; Weinberg A. M.; Prohaska T. Long-Term in Vivo Degradation Behavior and near-Implant Distribution of Resorbed Elements for Magnesium Alloys WZ21 and ZX50. Acta Biomater. 2016, 42, 440–450. 10.1016/j.actbio.2016.06.025. 27343708

[ref60] Krause A.; Von Der Höh N.; Bormann D.; Krause C.; Bach F. W.; Windhagen H.; Meyer-Lindenberg A. Degradation Behaviour and Mechanical Properties of Magnesium Implants in Rabbit Tibiae. J. Mater. Sci. 2010, 45 (3), 624–632. 10.1007/s10853-009-3936-3.

[ref61] Schauer A.; Redlich C.; Scheibler J.; Poehle G.; Barthel P.; Maennel A.; Adams V.; Weissgaerber T.; Linke A.; Quadbeck P. Biocompatibility and Degradation Behavior of Molybdenum in an in Vivo Rat Model. Materials 2021, 14 (24), 7776 10.3390/ma14247776. 34947370 PMC8705131

[ref62] Nagaraja S.; Di Prima M.; Saylor D.; Takai E. Current Practices in Corrosion, Surface Characterization, and Nickel Leach Testing of Cardiovascular Metallic Implants. J. Biomed. Mater. Res., Part B 2017, 105 (6), 1330–1341. 10.1002/jbm.b.33630. PMC6026850 26880035

[ref63] Høl P. J.; Gjerdet N. R.; Jonung T. Corrosion and Metal Release from Overlapping Arterial Stents under Mechanical and Electrochemical Stress – An Experimental Study. J. Mech. Behav. Biomed. Mater. 2019, 93, 31–35. 10.1016/j.jmbbm.2019.02.001. 30769231

[ref64] Cohen Hubal E. A.; Wetmore B. A.; Wambaugh J. F.; El-Masri H.; Sobus J. R.; Bahadori T. Advancing Internal Exposure and Physiologically-Based Toxicokinetic Modeling for 21st-Century Risk Assessments. J. Exposure Sci. Environ. Epidemiol. 2019, 29 (1), 11–20. 10.1038/s41370-018-0046-9. PMC6760598 30116055

[ref65] Sarigiannis D. A.; Karakitsios S.; Dominguez-Romero E.; Papadaki K.; Brochot C.; Kumar V.; Schumacher M.; Sy M.; Mielke H.; Greiner M.; Mengelers M.; Scheringer M. Physiology-Based Toxicokinetic Modelling in the Frame of the European Human Biomonitoring Initiative. Environ. Res. 2019, 172, 216–230. 10.1016/J.ENVRES.2019.01.045. 30818231

[ref66] Food and Drug Administration. Advancing Alternative Methods at FDA. https://www.fda.gov/science-research/about-science-research-fda/advancing-alternative-methods-fda (accessed 2023–12–18).

[ref67] European Commission. EU Reference Laboratory for alternatives to animal testing (EURL ECVAM). https://joint-research-centre.ec.europa.eu/eu-reference-laboratory-alternatives-animal-testing-eurl-ecvam_en (accessed 2023–12–18).

[ref68] Zia A. W.; Liu R.; Wu X. Structural Design and Mechanical Performance of Composite Vascular Grafts. Bio-des. Manuf. 2022, 5 (4), 757–785. 10.1007/s42242-022-00201-7.

[ref69] Croucher J.; Mahomed A. Concept and Simulation of an Alternative Design for an Orthopaedic Shoulder Implant. J. Med. Eng. Technol. 2022, 46 (1), 1–15. 10.1080/03091902.2021.1967489. 34549681

[ref70] Eshghi N.; Hojjati M. H.; Imani M.; Goudarzi A. M. Finite Element Analysis of Mechanical Behaviors of Coronary Stent. Procedia Eng. 2011, 10, 3056–3061. 10.1016/j.proeng.2011.04.506.

[ref71] Lopez-Perez A.; Sebastian R.; Ferrero J. M. Three-Dimensional Cardiac Computational Modelling: Methods, Features and Applications. BioMed. Eng. OnLine 2015, 14 (1), 1–31. 10.1186/s12938-015-0033-5. 25928297 PMC4424572

[ref72] Gray R. A.; Pathmanathan P. Patient-Specific Cardiovascular Computational Modeling: Diversity of Personalization and Challenges. J. Cardiovasc. Trans. Res. 2018, 11 (2), 80 10.1007/s12265-018-9792-2. PMC5908828 29512059

[ref73] Lee L. C.; Genet M.; Dang A. B.; Ge L.; Guccione J. M.; Ratcliffe M. B. Applications of Computational Modeling in Cardiac Surgery. J. Card. Surg. 2014, 29 (3), 293 10.1111/jocs.12332. 24708036 PMC4045706

[ref74] Food and Drug Administration. Reporting of Computational Modeling Studies in Medical Device Submissions; FDA, 2016.

[ref75] Saylor D. M.; Chandrasekar V.; Elder R. M.; Hood A. M. Advances in Predicting Patient Exposure to Medical Device Leachables. Med. Devices Sens. 2020, 3 (1), e10063 10.1002/mds3.10063.

[ref76] Saylor D. M.; Craven B. A.; Chandrasekar V.; Simon D. D.; Brown R. P.; Sussman E. M. Predicting Patient Exposure to Nickel Released from Cardiovascular Devices Using Multi-Scale Modeling. Acta Biomater. 2018, 70, 304–314. 10.1016/j.actbio.2018.01.024. 29408403

[ref77] Turner P.; Elder R. M.; Nahan K.; Talley A.; Shah S.; Duncan T. V.; Sussman E. M.; Saylor D. M. Leveraging Extraction Testing to Predict Patient Exposure to Polymeric Medical Device Leachables Using Physics-Based Models. Toxicol. Sci. 2020, 178 (1), 201–211. 10.1093/toxsci/kfaa140. 33111940

[ref78] Campbell J. L.; Clewell R. A.; Gentry P. R.; Andersen M. E.; Clewell H. J. Physiologically Based Pharmacokinetic/Toxicokinetic Modeling. In Computational Toxicology, Methods in Molecular Biology; Humana Press, 2012; pp 439–499 10.1007/978-1-62703-050-2_18. 23007440

[ref79] Mielke H.; Gundert-Remy U. Physiologically Based Toxicokinetic Modelling as a Tool to Support Risk Assessment: Three Case Studies. J. Toxicol. 2012, 2012, 359471 10.1155/2012/359471. 22649449 PMC3357559

[ref80] Sasso A. F.; Isukapalli S. S.; Georgopoulos P. G. A Generalized Physiologically-Based Toxicokinetic Modeling System for Chemical Mixtures Containing Metals. Theor. Biol. Med. Model. 2010, 7 (1), 17 10.1186/1742-4682-7-17. 20525215 PMC2903511

[ref81] Webb G. I.; Sammut C.; Perlich C.; Horváth T.; Wrobel S.; Korb K. B.; Noble W. S.; Leslie C.; Lagoudakis M. G.; Quadrianto N.; Buntine W. L.; Quadrianto N.; Buntine W. L.; Getoor L.; Namata G.; Getoor L.; Han X. J. J.; Ting J.-A.; Vijayakumar S.; Schaal S.; Raedt L. De. Leave-One-Out Cross-Validation. In Encyclopedia of Machine Learning; Springer: Boston, MA, 2011; pp 600–601 10.1007/978-0-387-30164-8_469.

