# Supplementary material for: A Predictive Toxicokinetic Model for Nickel Leaching from Vascular Stents
Source: ACS Biomater Sci Eng. 2024 Mar 25;10(4):2534–51. doi: 10.1021/acsbiomaterials.3c01436 (PMC11005016; doi:10.1021/acsbiomaterials.3c01436)
Supplement: Supplementary file 1 — ab3c01436_si_001.pdf [file ab3c01436_si_001.pdf]

SUPPORTING INFORMATION

A Predictive Toxicokinetic Model for Nickel Leaching from Vascular Stents

Matheos Giakoumi[1], Pavlos S. Stephanou[2], Despoina Kokkinidou[1], Chara Papastefanou[3], Andreas Anayiotos[1] and Konstantinos Kapnisis[1,*]

[1] Department of Mechanical Engineering and Materials Science and Engineering, Cyprus University of Technology, Limassol 3036, Cyprus
[2] Department of Chemical Engineering, Cyprus University of Technology, Limassol 3036, Cyprus
[3] Cp Foodlab Ltd, Nicosia 2326, Cyprus

* Corresponding author: k.kapnisis@cut.ac.cy

A. Device release model: parameters' optimization

The device's cumulative nickel release parameters, α and τ as designated in Eq. (1), were fine-tuned with the use of MATLAB's [1] Multistar global optimization object, in conjunction with the local optimization tool, lsqcurvefit, which employs the Levenberg-Marquardt algorithm. The optimization process was regulated by an objective function, defined as the sum of the squared difference between the model's predicted cumulative nickel release and the measured release data, with convergence tolerance of 10^{-5}. The optimized values of α and τ for the three nitinol stents, tested and characterized in section 2.2, are listed in Table S1.

Table S1: *Fitting of in vitro nickel release measurements from stents reported in section 2.2. to Eq. (1).*

Stent Type	Fit using Eq. (1)	
	α $\left(\frac{\mathrm{ng}}{\mathrm{mm}^2}\right)$	τ (day)
**V1HT**	38.7	18.7
**V1EP**	29.3	21.1
**V2EP**	29.2	43.3

Figure S1 presents the best-fit curve using the optimum values of α and τ (Table S1), normalized with respect to the surface area, versus the *in vitro* nickel release measurements from the three devices, as outlined in section 2.2.

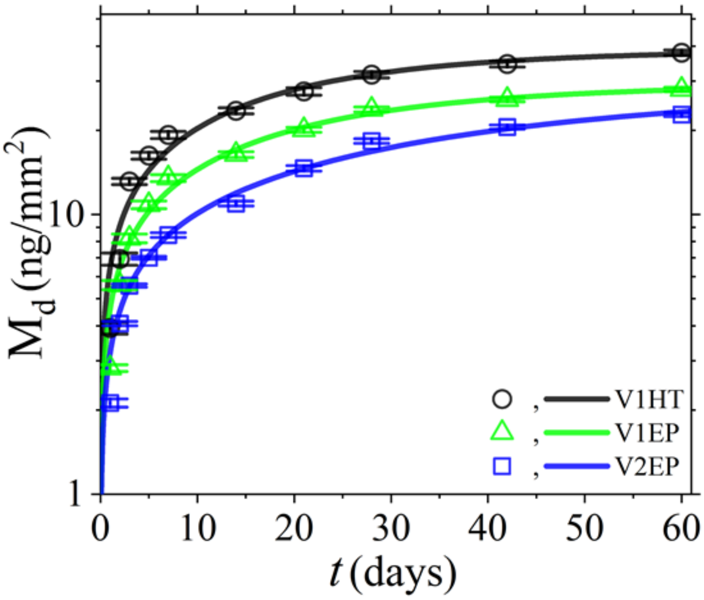

Figure S1: Fitting Eq. (1) to in vitro immersion data reported in section 2.2. The figure shows the representative cumulative nickel release measurements normalized by the surface area (in units of ng/mm^2*), and the corresponding best-fit curves (lines) against the in vitro experimental data (symbols).*

B. PBTK Model: parameters' optimization

The biokinetic parameters of the PBTK model, outlined in Eqs. (3), were separately optimized for the V1HT & V1EP devices via the patternsearch global optimization algorithm [1]. This algorithm was chosen due to the non-smoothness of the adapted single-objective function defined by each compartment using available *in vivo* data. Rather than a single run, the optimal values were derived from a series of iterations by which only the most important kinetic rates were considered as time-dependent, beginning with various initial guesses and characteristics of the algorithm (like mesh tolerance, and convergence tolerance). Moreover, the gspositivebasins2n poll method was implemented in conjunction with the Complete Poll & Search feature in MATLAB [1] to improve the algorithm's performance metrics.

The method to determine the objective function for each device is based on the approach outlined in Section 2.6, and the weight factor values relied on accessible *in vivo* data. Nearly all the weight factors were computed via the equalities given in Eqs. (11), as most of the compartments had only one piece of data that violated the conditions. Nevertheless, for the local tissue compartment in the V1HT device and the liver compartment in V1EP, two data points violated the conditions of Eqs. (10), necessitating the use of an optimization process to obtain σ_ι parameter needed in Eqs. (11). The fminsearch optimization function was employed for this optimization. Note that the urine compartment is not subjected to these conditions due to unavoidable uncertainty in experimental measurements. Table S2 shows the resultant weight factors corresponding to the devices V1HT and V1EP.

Table S2: *Weight factors indicating the confidence in the experimental in vivo data for the devices V1HT and V1EP. These weight factors define the objective function to be used for the optimization of the PBTK model [Eqs. (3)] for each device.*

**V1HT**	w_{ij}			
i/j	**Control**	**28 days**	**56 days**	**126 days**
**Local Tissues**	0	0.514	0.227	0.259
**Blood**	0	0.743	0.176	0.082
**Liver**	0	0.163	0.177	0.659
**Brain**	0	0.333	0.333	0.333
**Lungs**	0	0.335	0.335	0.330
**Kidneys**	0	0.490	0.490	0.002
**Urine**	0	0.333	0.333	0.333
**V1EP**				
**Local Tissues**	0	0.333	0.333	0.333
**Blood**	0	0.360	0.360	0.280
**Liver**	0	0.155	0.197	0.648
**Brain**	0	0.360	0.280	0.360
**Lungs**	0	0.341	0.341	0.319
**Kidneys**	0	0.307	0.576	0.117
**Urine**	0	0.333	0.333	0.333

During the optimization process, the parameters constituting the objective function $\left(w_{ij}\ \&\ P\right)$ were held constant. What varied between the iterations were only the lower and upper boundaries of the kinetic rates' parameters and the specifications of the algorithm. The resulting fits for the V1HT and V1EP devices are depicted in Figures S2 and S3, respectively, and the RMSE of each compartment is presented in Table S3.

V1HT

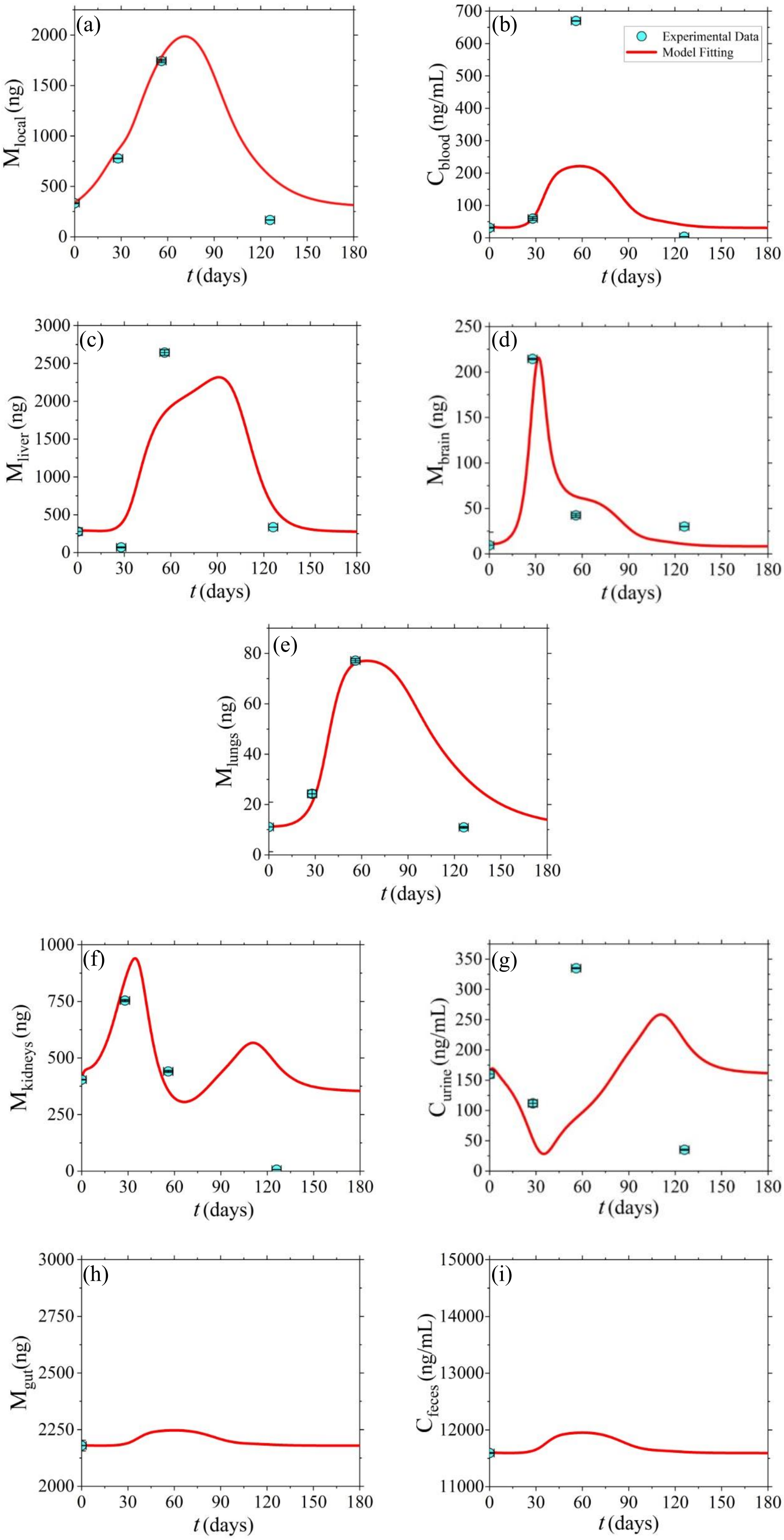

***Figure S2**: Fitting of Eqs. (3) against the in vivo data of device V1HT.*

V1EP

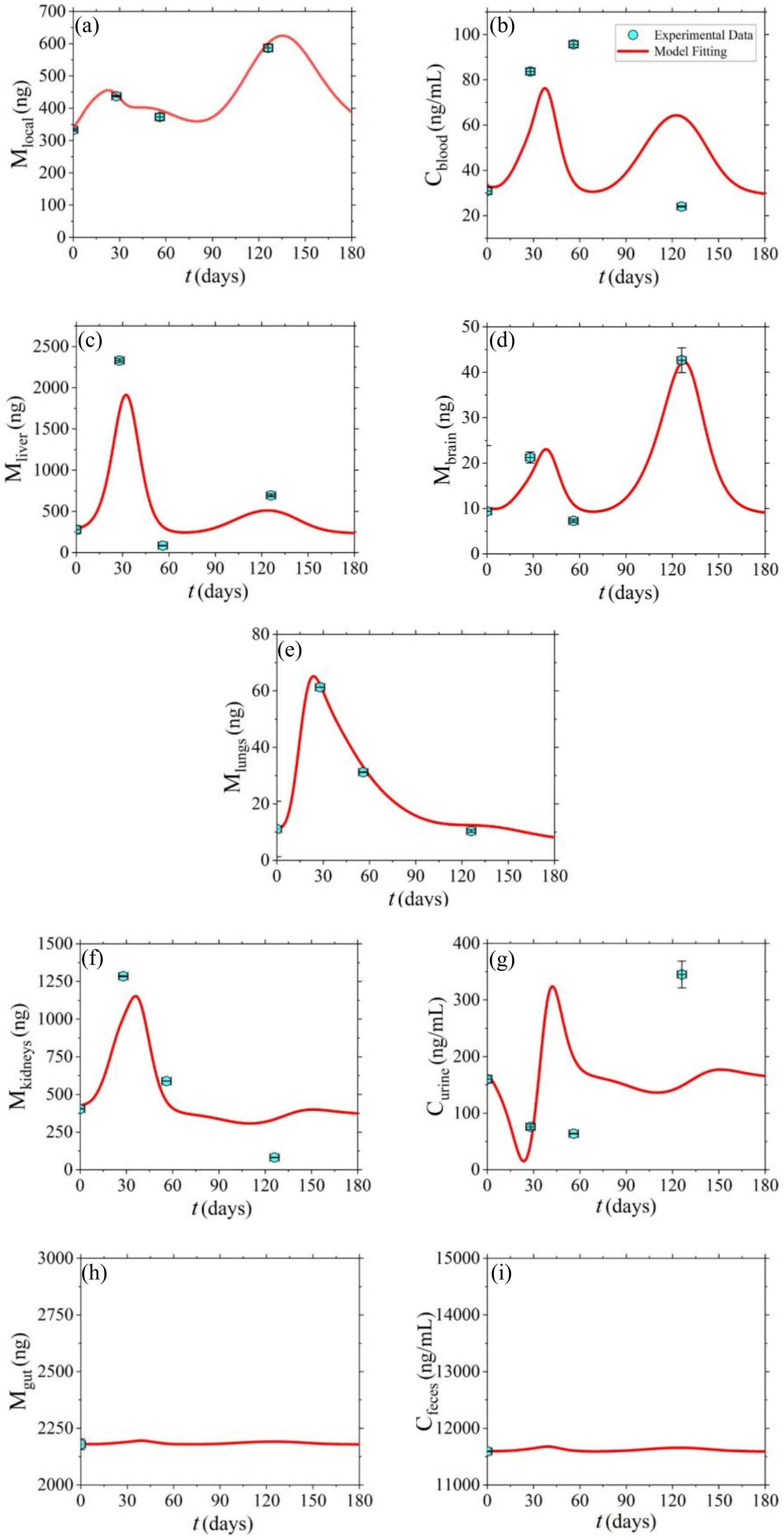

Figure S3: *Fitting of Eqs. (3) against the in vivo data of device V1EP.*

Table S3: Root Mean Square Error of each compartment ($RMSE_i$) for the fitting results of the devices V1HT and V1EP.

Compartment	$RMSE_i$ [eq. (12)]	
	V1HT	V1EP
Local Tissues (ng)	60.7	4.3
Blood (ng)	134.3	44.5
Liver (ng)	117.8	79.8
Brain (ng)	9.2	0.9
Lungs (ng)	3.4	0.5
Kidneys (ng)	30.8	59.1
Urine (ng)	7.8	6.0
$\frac{1}{P}\sum RMSE_i$ [eq. (8)]	52.0	27.9

C. Toxicological Risk Assessment – Device release rate violation

In section 3.2, a toxicological risk assessment was conducted by evaluating the violation of the compartmental TI_i values, as defined in section 2.7, by the predicted release rate profiles (dM_i/dt) for the V1HT, V1EP and V2EP devices. In Figure S4, the time-depended device release-rate $(\dot{M}_d)$ is compared against the total TI value. The figure indicates that for all devices the predefined threshold is exceeded, with the level and timespan of the violations decreasing between the V1HT, V1EP and V2EP devices.

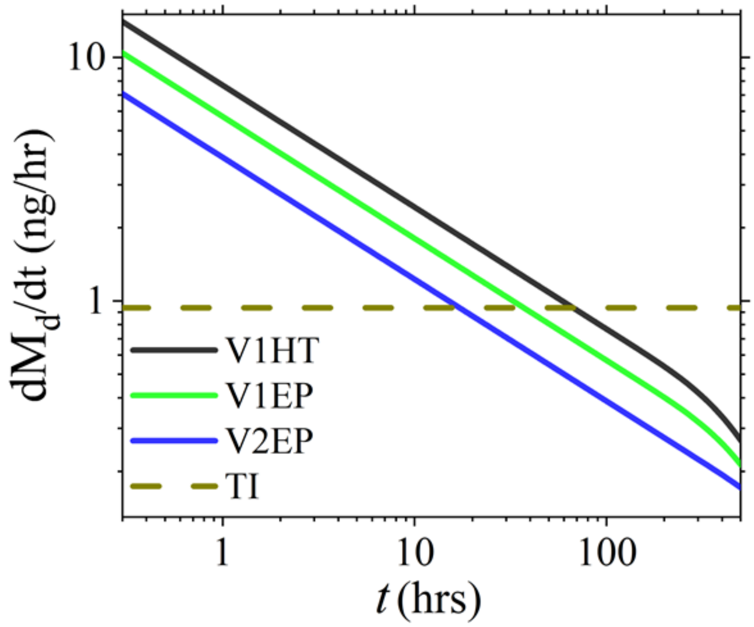

***Figure S4**: Tolerable Intake (TI) and device release rate (dM_d/dt) for V1HT, V1EP and V2EP devices.*

D. Lumping and Splitting

The execution of *in vivo* experiments is a laborious and costly procedure. As such, in many studies, only a limited number of samples are analyzed, with primary emphasis given to obtaining samples from blood and or excrement. In other cases, tissues, organs, or body fluids that are pharmacokinetically and toxicologically similar can be lumped/grouped together. In such an approach,

the model development starts on the version that has the finer information, and decisions are made to coarse grain some elements (tissues and body fluids) together, provided this can be justified. In this section, we are presenting the lumping and splitting of the PBTK model, described in Figure 1 and Eqs. (3) in section 2.5.2 (Biokinetics), as discussed in section 4 (Discussion).

D1. Lumping

In our first approach, the aim is to start from a finer level of model description and proceed to "simplify" it by lumping similar tissues. Thus, our starting point is the evolution equations of the new PBTK model, Eqs. (3). We note that the evolution equations for the tissues that exchange Ni (or other substances or particles) with blood only, i.e., brain, lungs, and liver, can be written in the form:

$$\frac{dM_i}{dt} = k_{\mathrm{ibl}}(t) M_{\mathrm{bl}}(t) - k_{\mathrm{ibl}}(t) M_i(t) \tag{S1}$$

Here, $i = \{\mathrm{br}, \mathrm{lu}, \mathrm{liv}\}$. To lump these similar tissues into a single compartment, called "other tissues", the total Ni mass within this compartment must be the sum of the Ni mass of its corresponding components, that is:

$$M_{\mathrm{ot}}(t) \equiv \sum_{i=\mathrm{br,lu,liv}} M_i(t) \tag{S2}$$

Thus, the evolution equation for this new compartment must be completely dictated by the evolution equations of its constituent tissues:

$$\frac{dM_{\mathrm{ot}}(t)}{dt} = \sum_{i=\mathrm{br,lu,liv}} \frac{dM_i(t)}{dt} = - \sum_{i=\mathrm{br,lu,liv}} k_{\mathrm{ibl}}(t) M_i(t) + \left(\sum_{i=\mathrm{br,lu,liv}} k_{\mathrm{bli}}(t) \right) M_{\mathrm{bl}}(t) \tag{S3}$$

When forcing this to match the evolution equation that would be proposed in the coarser level of description, namely:

$$\frac{dM_{ot}}{dt} = -k_{\mathrm{otbl}}(t) M_{\mathrm{ot}}(t) + k_{\mathrm{blot}}(t) M_{\mathrm{bl}}(t) \tag{S4}$$

the following equalities/definitions must hold:

$$k_{\mathrm{otbl}}(t) M_{ot}(t) = \sum_{i=\mathrm{br,lu,liv}} k_{\mathrm{ibl}}(t) M_i(t) \Rightarrow$$

$$k_{\mathrm{otbl}}(t) = \sum_{i=\mathrm{br,lu,liv}} k_{i\mathrm{bl}}(t) \frac{M_i(t)}{M_{\mathrm{ot}}(t)} = \sum_{i=\mathrm{br,lu,liv}} k_{i\mathrm{bl}}(t) y_i(t) \tag{S5a}$$

$$k_{\mathrm{blot}}(t) = \sum_{i=\mathrm{br,lu,liv}} k_{\mathrm{bli}}(t) \tag{S5b}$$

Thus, the kinetic rate describing the exchange of Ni from the "other tissues" compartment toward the blood $k_{\mathrm{otbl}}(t)$ must be the weighted average of the respective kinetic rates, with the weight being the average mass fraction of each tissue in the "other tissue" compartment. Since we start from the finer level of model description both are known. Finally, the kinetic rate that describes the reverse

exchanges, i.e., from blood to the "other tissues" compartment, k_{blot}, is conveniently equal to the sum of the respective kinetic rates.

Then, Equations (3) (in section 2.5.2) become:

$$\frac{\mathrm{d}M_{\mathrm{lt}}}{dt} = \left(1 - F(t)\right)\dot{M}_d(t) + k_{\mathrm{bllt}}(t)M_{\mathrm{bl}}(t) - k_{\mathrm{ltbl}}(t)M_{\mathrm{lt}}(t) \quad \text{(S6a)}$$

$$\frac{dM_{\mathrm{bl}}}{dt} = F(t)\dot{M}_d(t) + k_{\mathrm{ltbl}}(t)M_{\mathrm{lt}}(t) + k_{\mathrm{otbl}}(t)M_{\mathrm{ot}}(t) + k_{\mathrm{kbl}}(t)M_k(t) - \left(k_{\mathrm{blot}}(t) + k_{\mathrm{bllt}}(t) + k_{\mathrm{blk}}(t) + k_{\mathrm{blg}}(t)\right)M_{\mathrm{bl}}(t) + k_{\mathrm{gbl}}(t)M_g(t) \quad \text{(S6b)}$$

$$\frac{dM_{\mathrm{ot}}}{dt} = k_{\mathrm{blot}}(t)M_{\mathrm{bl}}(t) - k_{\mathrm{otbl}}(t)M_{\mathrm{ot}}(t) \quad \text{(S6c)}$$

$$\frac{dM_k}{dt} = k_{\mathrm{blk}}(t)M_{\mathrm{bl}}(t) - \left(k_{\mathrm{kbl}}(t) + k_u(t)\right)M_k(t) \quad \text{(S6d)}$$

$$\frac{dM_g}{dt} = k_{\mathrm{blg}}(t)M_{\mathrm{bl}}(t) - \left(k_{\mathrm{gbl}}(t) + k_f(t)\right)M_g(t) + k_{\mathrm{diet}}(t) \quad \text{(S6e)}$$

$$\frac{dM_u}{dt} = C_u(t)Q_u = k_u(t)M_k(t) \quad \text{(S6f)}$$

$$\frac{dM_f}{dt} = C_f(t)Q_f = k_f(t)M_g(t) \quad \text{(S6g)}$$

Overall, it can be concluded that lumping is feasible (if required and justified).

D2. Splitting

During splitting, our approach starts from the coarser level of model description, and we wish to split a more general compartment, such as the "other tissues" compartment in our PBTK model, into some or all its constituents. In other words, we aim to start with Eqs. (S6) and obtain, rigorously, Eqs. (3) of the new PBTK model. Again, equations (S5) above need to hold but the unknowns in this case are both the masses $M_i(t)$ [or equivalently the mass fractions $y_i(t)$] of each constituent compartment and the kinetic rates between each constituent compartment and blood. Without making any further assumptions, it is obvious that splitting cannot be applied in this setting. Even if the average mass fractions, $\langle y_i \rangle$, (see Sec. 2.8 of the main text) of each compartment in the "other tissue" compartment were known, the system of equations is underdetermined. Unless further assumptions are made, e.g., providing physiologically relevant correlations between the unknown kinetic rates, splitting the PBTK model is not possible.

References

[1] The MathWorks Inc, MATLAB version: 9.14.0.2239454 (R2023a), (2023). https://www.mathworks.com/.
